# APOBEC3-driven attenuation of the 2022 global outbreak monkeypox virus relative to its clade IIb ancestor

**DOI:** 10.1038/s41467-026-76580-7

**Published:** 2026-08-11

**Authors:** Rebecca P. Sumner, Lucy Eke, Bruno Hernáez, Alasdair J. M. Hood, Telma Sancheira Freitas, Hannah Ashby, Ailish Ellis, Marine Petit, Sian Lant, Isobel Stokes, Preetam Parija, Isabel Alonso, Francisco J. Alvarez-de Miranda, Alazne R. Unanue, Kevin Maringer, Isabelle Dietrich, Bryan Charleston, Geoffrey L. Smith, David O. Ulaeto, Antonio Alcamí, Carlos Maluquer de Motes

**Affiliations:** 1 Department of Microbial Sciences, University of Surrey, Guildford, UK; 2 Department of Infectious Diseases, King’s College London, London, UK; 3 Centro de Biología Molecular Severo Ochoa, Consejo Superior de Investigaciones Científicas (CSIC)-Universidad Autónoma de Madrid (UAM), Madrid, Spain; 4 The Pirbright Institute, Pirbright, UK; 5 Sir William Dunn School of Pathology, University of Oxford, Oxford, UK; 6 CBR Division, Defence Science and Technology Laboratory, Salisbury, UK

**Keywords:** Pox virus, Viral host response, Viral immune evasion, Viral evolution

## Abstract

Monkeypox virus (MPXV) is a zoonotic virus endemic to Africa that has recently re-emerged, becoming the first orthopoxvirus with sustained human-to-human transmission since smallpox eradication. The 2022 global epidemic of MPXV was caused by Clade IIb virus (lineage B.1) that derived from the lineage A endemic to West Africa through APOBEC3 microevolution^1–3^. Here, comparison of lineage B.1 virus with its closest earlier A.1 ancestor reveals that despite only 46 nucleotide differences, lineage B.1 has attenuating phenotypic changes. Both viruses replicated equivalently in cell culture, but B.1 had defects in long-range spread, which mapped to APOBEC3 mutation E353K in OPG057 (F13L). MPXV B.1 was also defective in innate immune control, inducing higher IFNβ and innate immune signalling gene expression, and showing reduced capacity to suppress interleukin-1β-mediated responses, which mapped to APOBEC3 mutation R48C in OPG047 (F3L). Finally, MPXV B.1 had reduced virulence in a mouse model of MPXV infection relative to the ancestral A.1 strain. Our work demonstrates that the 2022 global outbreak MPXV was intrinsically attenuated, with attenuating mutations that are absent in concurrent, more severe clade IIb outbreaks. Our data also reveal that APOBEC3 editing is a mechanism of MPXV phenotypic variation, potentially contributing to clade evolution and competition.

## Abstract (web summary)

Here, the authors compare the ancestral MPXV A.1 and 2022 outbreak lineage B.1. They show that APOBEC3-driven mutations attenuate viral spread, immune evasion and virulence of B.1. Despite similar cell-culture replication, B.1 was less pathogenic in vivo, highlighting APOBEC3 as a potential driver of MPXV evolution.

## Introduction

Monkeypox virus (MPXV) is an orthopoxvirus (OPXV) traditionally confined to West (Clade IIa and IIb) and Central (Clade Ia, Ib) Africa, where it infects several rodent species. MPXV causes mpox in humans, a rash and systemic disease similar to smallpox. Historical data report inefficient spread of MPXV and low secondary attack rates even within household contacts^4,5^, supporting the notion that MPXV is poorly adapted to human-human transmission and outbreaks in endemic areas are sustained by periodic re-introductions from the animal reservoir. From May 2022, however, >100,000 cases of mpox were detected outside the African continent, mostly concentrated in the gay, bisexual and men-who-have-sex-with-men communities, resulting in the declaration of a public health emergency of international concern by the WHO in July 2022. Little is known about how MPXV (and OPXV in general) emerge and interact with the human host, particularly with regards to innate immunity, which is a critical barrier to zoonotic infection^6–8^. Aside from sporadic infections with cowpox virus, the only human OPXV disease, smallpox (caused by variola virus, VARV) was eradicated >40 years ago and the mechanisms by which it originally emerged are not clear^9^.

Phylogenetic analyses indicate that the Clade IIb MPXV strain that caused the 2022 global outbreak (termed lineage B) is descended from Clade IIb lineage A.1^1–3^ reported from an outbreak occurring in Nigeria between September 2017 and September 2018^10^ and derives from the same zoonotic event^1,3^. Clinical and epidemiological differences exist between lineage B and lineage A strains, with the 2022 global lineage B outbreak being largely sustained by sexual contact transmission and presenting with novel clinical manifestations, including a significant primary rash at the infection site, reduced number of lesions in the secondary disseminated rash, and lower case-fatality rate (CFR)^11,12^. Lineage A viruses have established continuous human-to-human transmission in endemic areas^2^. Conversely, lineage B has established an unprecedented global transmission web amongst humans affecting >110 countries. Whilst available clinical data indicate that Clade IIb is the mildest mpox infection, a recent outbreak in Sierra Leone (2025) derived from Nigerian lineage A viruses has shown unexpectedly high virulence closely resembling more virulent Clade I strains^13,14^. It remains unclear, however, whether these differences are solely explained by non-biological factors or, conversely, distinguishable properties between clade IIb viruses exist.

Lineage B isolates from the first 2022 cases revealed 46 unique single-nucleotide changes distributed among them, differentiating them from A.1 isolates^1,15^. This equates to a mutation rate of ~6 changes per year, much higher than expected for a virus estimated to have ~0.35 nucleotide substitutions per year^16^. Remarkably, these changes are not replication errors, but are consistent with cytosine deaminase activity, likely from host apolipoprotein B mRNA editing enzyme catalytic subunit 3 (APOBEC3) enzymes^3^ and are inherited in the descendant diversity encompassing all known lineage B viruses. More recently, APOBEC3-like mutations have also been reported in a Clade Ib MPXV strain spreading in Central Africa^17,18^. Whilst the APOBEC3-like mutational footprint represents a signature of MPXV transmission amongst humans^3^, it remains unclear whether these mutations impact virus fitness and interactions with the host. Here, we took a comparative virology approach to study viral replication and host innate responses to MPXV infection and to determine the biological consequences of the mutational footprint that distinguishes clade IIb lineage A and B MPXV strains.

## Results

### MPXV Clade IIb lineage B is defective for viral spread

**Fig. 1:**
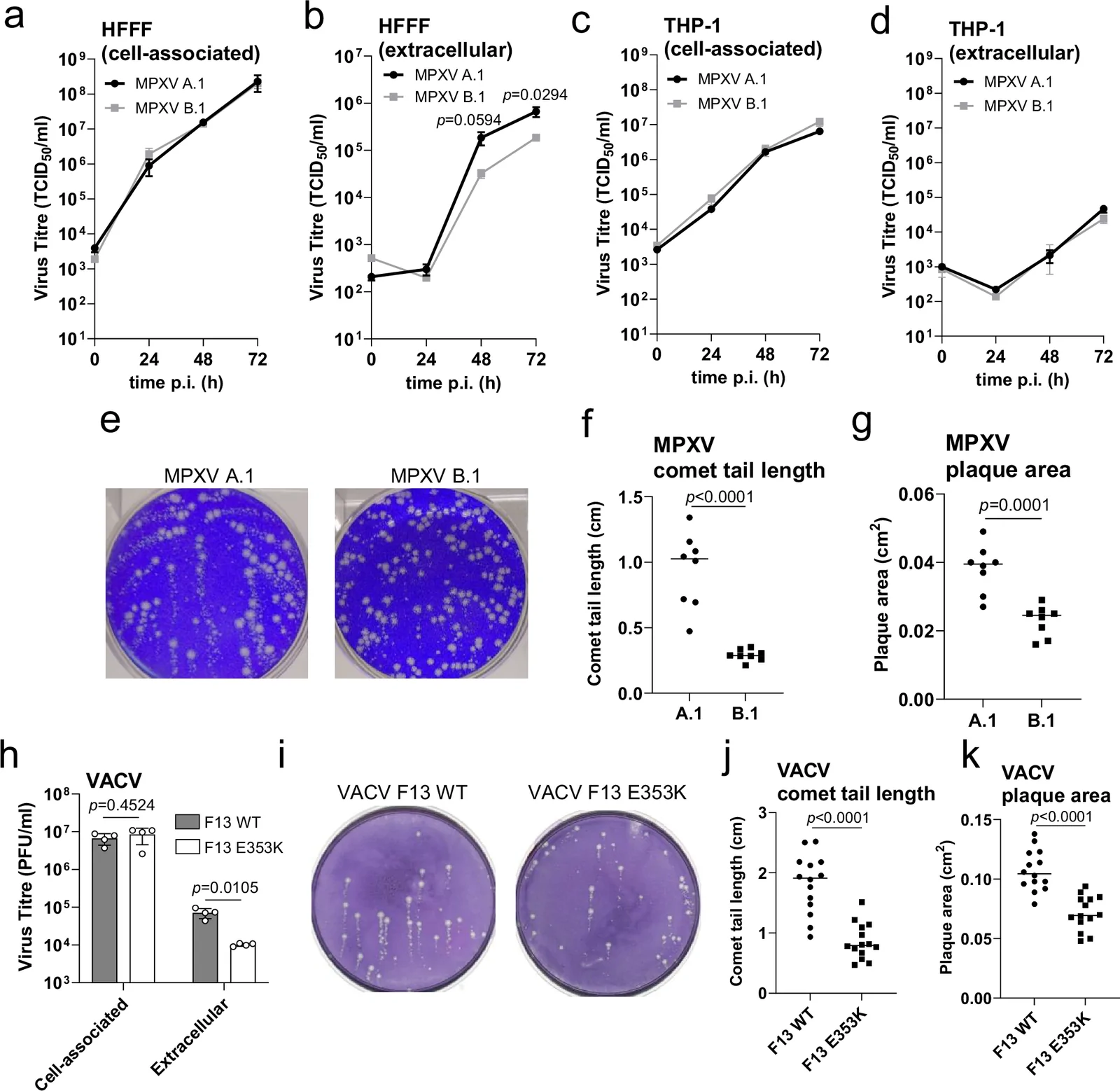
MPXV Clade IIb lineage B.1 has a defect in viral spread. **a**–**d** Monkeypox virus (MPXV) A.1 and B.1 replication over 72 h in human foetal foreskin fibroblasts (HFFF) (**a**, **b**) or THP-1-derived macrophages (**c**, **d**) infected with 0.05 p.f.u. per cell quantifying cell-associated (**a**, **c**) and extracellular virus (**b**, **d**). *n* = 3 biological replicates per condition. Data are mean ± SD. Statistical analyses were performed using an unpaired two-sided Student’s *t*-test. **e** Representative comet tail assay from BSC-40 cells infected with MPXV A.1 or B.1 for 96 h with a liquid overlay. **f**, **g** Quantitation of comet tail length (**f**) and plaque area (**g**) from (**e**). *n* = 8 individual comet tails or plaques per condition from a single experiment. The horizontal bar represents the median. Statistical analyses were performed using an unpaired two-sided Student’s* t*-test. **h** Vaccinia virus (VACV) F13 WT and VACV F13 E353K cell-associated and extracellular virus titres at 48 h from BSC-40 cells infected with 0.005 p.f.u. per cell. *n* = 4 biological replicates per condition. Data are mean ± SD. Statistical analyses were performed using an unpaired two-sided Student’s *t*-test. **i** Representative comet tail assay from BSC-40 cells infected with VACV F13 WT or VACV F13 E353K for 72 h with a liquid overlay. **j**, **k** Quantitation of comet tail length (**j**) and plaque area (**k**) from (**i**). *n* = 14 individual comet tails or plaques per condition from a single experiment. The horizontal bar represents the median. Statistical analyses were performed using an unpaired two-sided Student’s *t*-test. All data presented are representative of at least 2 experimental repeats. **e** is representative of 3 repeats. Source data are provided as a Source Data file.

In humans, most OPXV infect and/or transmit through direct contact with body fluids and skin lesions, where the virus initially infects keratinocytes and fibroblasts before disseminating within the body through immune cells such as migratory inflammatory monocytes^19,20^. We therefore assessed viral replication following MPXV infection of primary human fibroblasts (HFFF) and THP-1-derived macrophages infected with either Clade IIb, lineage B.1 (isolate CVR_S1) or Clade IIb, lineage A.1 (isolate UK_P3) viruses. OPXV exist in two infectious forms: intracellular mature virus (IMV), a cell-associated form that accumulates inside the cell and is released upon cell lysis, and extracellular enveloped virus (EEV) that are released for dissemination and cell-to-cell spread within the host^20^. Titres of cell-associated virus were comparable between MPXV A.1 and B.1 over 72 h growth curves in primary fibroblasts (Fig. 1a), but the extracellular virus titre upon MPXV B.1 infection was slightly lower, reaching statistical significance at 72 h (Fig. 1b). In THP-1-derived macrophages, cell-associated virus titre was also equivalent, but EEV titres were low for both (Fig. 1c, d). Consistent with HFFF data, MPXV A.1 and B.1 plaque size was equivalent in BSC-40 cells using a semi-solid overlay (Supplementary Fig. 1a, b), but reduced dissemination by B.1 was observed by “comet tail” assay under liquid overlay, where secondary long-range spread of virus and plaque area was greater for MPXV A.1 (Fig. 1e–g and Supplementary Fig. 1c). We also repeated these experiments in the presence of ruxolitinib, an inhibitor of the Janus kinase/signal transducer and activator of transcription (JAK/STAT) pathway, to account for the potential activation of an interferon (IFN) response by B.1 (see below) suppressing virus spread. Ruxolitinib treatment did not rescue EEV titre or comet tail formation by B.1 (Supplementary Fig. 1d and f) despite being able to suppress IFN activity in both HFFF and BSC-40 cells (Supplementary Fig. 1e and g). Differences in viral spread at least partially mapped to the E353K coding change in MPXV B.1 OPG057 (Supplementary Fig. 2 and Supplementary Table 1), encoding the highly conserved protein F13 with essential roles in EEV formation^21^, as engineered vaccinia virus (VACV) carrying this mutation had reduced EEV, but not IMV titre (Fig. 1h), and had defects in comet tail formation (Fig. 1i–k). The E-to-K change at position 353 did not affect F13 expression levels during MPXV infection (Supplementary Fig. 3). Lower extracellular virus titre had been reported for MPXV B.1 relative to Clade I and Clade IIa zoonotic isolates^22^. Our data demonstrates that this is not a general feature of Clade IIb viruses, because Clade IIb lineages A and B viruses differ in this respect, driven by APOBEC3-like mutation.

### Innate immune sensing of MPXV Clade IIb lineages

We next investigated innate immune control by MPXV. Host innate immunity is an important barrier to zoonotic infection, contributes to viral clearance and shapes adaptive immune responses. As cytosolic DNA replicating viruses, poxviruses are susceptible to detection by DNA sensor cyclic 2′3-GMP-AMP (cGAMP) synthetase (cGAS), leading to the production of a second messenger molecule (cGAMP) that binds stimulator of IFN genes (STING) and activates downstream innate transcription factors, including IFN responsive factor (IRF)3, to induce IFN expression and subsequent activation of the JAK/STAT pathway^23^. To study host innate responses to the Clade IIb A and B MPXV lineages, viruses were purified by sucrose density ultracentrifugation to minimise carryover of host cell proteins and debris, and experiments were performed at high multiplicity of infection (MOI) to negate differences in viral spread. Both viruses were grown in the same cells at the same time in parallel and tested for protein to infectivity ratios and these were found to be indistinguishable. Hallmarks of cGAS-STING activation, including phosphorylation of STING, IRF3, and STAT1, were not observed following MPXV A.1 or B.1 infection in either THP-1-derived macrophages (Supplementary Fig. 4a) or in primary fibroblasts (Supplementary Fig. 4b) up to 24 h post-infection, indicating evasion of this pathway. Phosphorylation of these proteins was observed following 2′3′-cGAMP transfection as expected (Supplementary Fig. 4a, b). Similar results were observed with other OPXV, including rodent-restricted ectromelia virus (ECTV) and more promiscuous species like cowpox virus (CPXV) and VACV that are able to infect humans but do not show sustained human-to-human transmission (Supplementary Fig. 4a, b), in agreement with previous reports^24^. Infection with an ECTV lacking the major DNA-sensing antagonist viral Schlafen (ECTVΔvSlfn) did, however, induce pathway activation as expected^25^. Furthermore, both MPXV strains suppressed STING/IRF3 phosphorylation following exogenous stimulation of infected cells with a dsRNA mimic (poly(I:C)), herring testis (HT)-DNA or 2′3′-cGAMP (Supplementary Fig. 4c), and were equally capable of suppressing HT-DNA- and poly(I:C)-induced *IFNβ* expression by qPCR (Supplementary Fig. 4d). Equivalent infection levels were monitored by blotting for the highly conserved OPXV protein OPG079 (Supplementary Fig. 4a–c). Our data demonstrate that both lineage A and B maintain the ability to inhibit this aspect of host innate immunity.

### MPXV induces a unique inflammatory response that is not observed with other OPXV

**Fig. 2:**
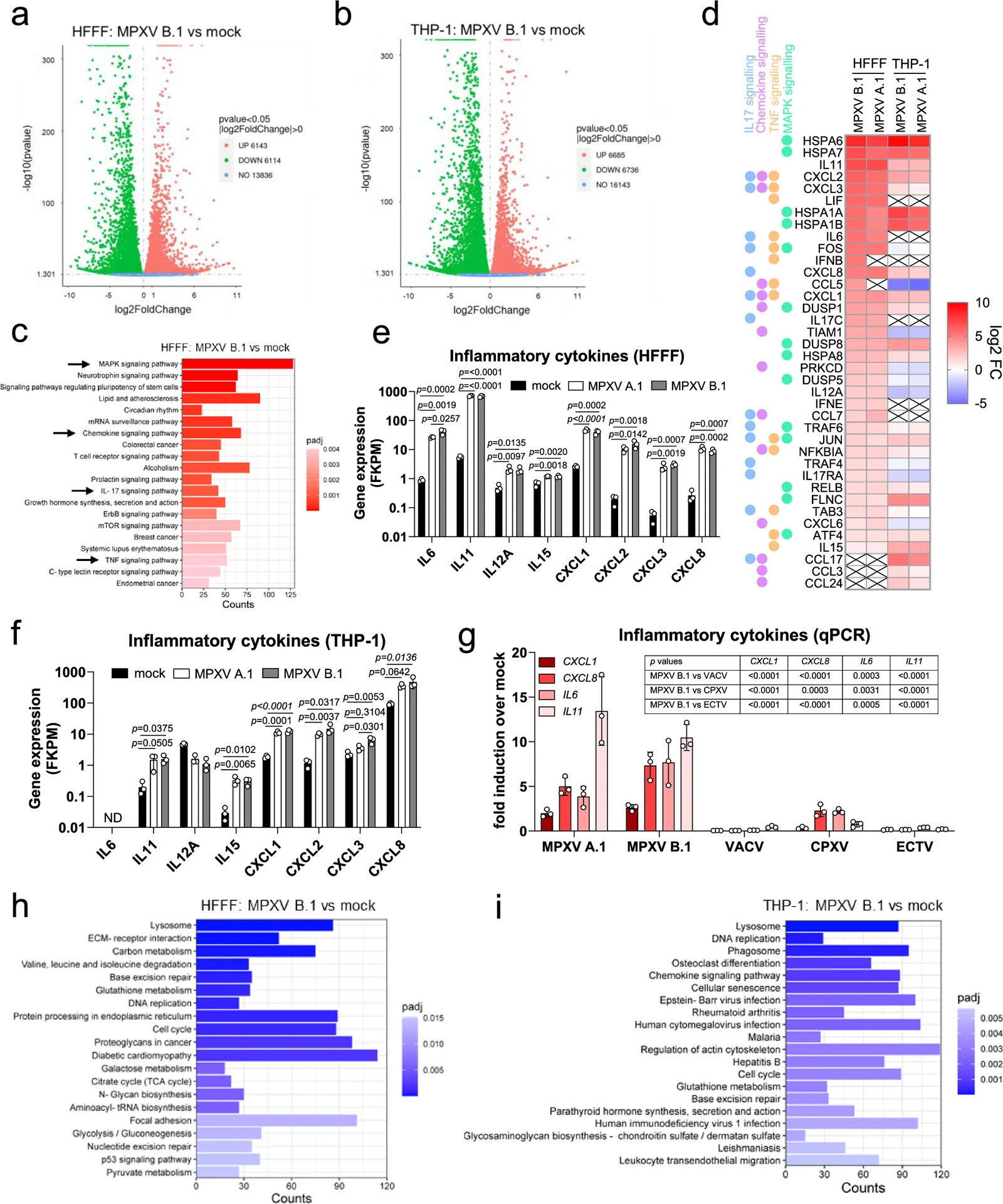
MPXV induces a unique inflammatory response in human cells. **a**, **b** Significantly differentially expressed genes (log2FC > 0.0) at 24 hpi by volcano plot analysis in human foetal foreskin fibroblasts (HFFF) (**a**) or THP-1-derived macrophages (**b**) infected with 5 p.f.u. per cell of monkeypox virus (MPXV) B.1, compared with the mock. *P* value determined by DESeq2. **c** Top 20 significantly upregulated pathways (KEGG analysis) from RNAseq data of HFFF cells infected with 5 p.f.u. per cell of MPXV B.1 for 24 h compared with mock-infected cells. *P* value determined by DESeq2. Pathways of interest are highlighted with a black arrow. **d** Heat map showing log2 fold change (FC) of selected significantly regulated genes from RNAseq data of HFFF or THP-1-derived macrophages infected for 24 h with ﻿5 p.f.u. per cell of MPXV A.1 or MPXV B.1. Boxes with an X indicate transcript not detected. The KEGG pathways to which these genes belong to are indicated on the left. **e**, **f** Expression level (FKPM) of a panel of inflammatory cytokines identified in (**d**) in HFFF (**e**) or THP-1-derived macrophages (**f**). Data are mean ± SD. *n* = 3 biological replicates per condition. Statistical analyses were performed per gene using an ordinary one-way ANOVA with Tukey’s multiple comparisons test. **g** RT-qPCR of selected inflammatory cytokines at 24 hpi from HFFF infected with 5 ﻿p.f.u. per cell of the indicated viruses. Data are mean ± SD. *n* = 3 biological replicates per condition. Statistical analyses were performed using an ordinary one-way ANOVA with multiple comparisons per gene. **h**, **i** Top 20 significantly downregulated pathways (KEGG analysis) from RNAseq data of HFFF (**h**) or THP-1-derived macrophages (**i**) infected with MOI 5 MPXV B.1 for 24 h compared with mock-infected cells. *P* value determined by DESeq2. All data are from a single RNA-seq experiment except (**g**), which is representative of at least 3 experimental repeats. Source data are provided as a Source Data file.

The ability of MPXV to cause a disseminated systemic infection and human-to-human transmission is a clear difference from other zoonotic OPXV. To gain a global overview of host responses to MPXV lineages, we performed total RNA sequencing in HFFF and THP-1-derived macrophages infected for 24 h at high MOI. MPXV induced large changes in RNA transcript levels, with around 12–13,000 differentially expressed genes (DEGs, >log2FC 0 and *P* < 0.05) with both strains and in both cell types (Fig. 2a, b and Supplementary Fig. 5a, b). One of the top upregulated KEGG pathways in response to MPXV infection in fibroblasts was MAPK signalling (Fig. 2c and Supplementary Fig. 5c). Both MPXV strains induced elevated expression of a variety of genes associated with this pathway in HFFF and THP-1-derived macrophages, including heat shock proteins (e.g. HSPA6, HSPA7, HSPA1A), dual-specificity phosphatases (e.g. DUSP1, DUSP8) and MAPK transcription factors (Fos, Jun), with a trend for greater induction by MPXV B.1 (Supplementary Fig. 6a–c). Upregulation of this pathway has previously been reported for other OPXV^26,27^ and in MPXV-infected human skin organoids^28^. We confirmed MAPK pathway activation (phosphorylated ERK, JNK, and p38) and protein induction (DUSP1) by MPXV A.1 and B.1 by immunoblotting (Supplementary Fig. 6d). Pharmacological inhibition of this pathway using activator protein (AP)−1 inhibitor SR11302 strongly curbed MPXV replication, in line with previous observations with VACV^26^ (Supplementary Fig. 6e).

In addition, MPXV induced the expression of genes belonging to various cytokine-related pathways, including chemokine, TNF and IL-17 signalling (Fig. 2c and Supplementary Fig. 5c). Infection with either lineage B or lineage A MPXV strains resulted in the elevated expression of a range of inflammatory cytokines belonging to these pathways, including ILs 6, 12A and 15 and CXCLs 1–3 and 8 (IL-8), particularly in primary fibroblasts (Fig. 2d, e), but also in THP-1-derived macrophages (Fig. 2d, f). Concomitantly, significant upregulation of fibrotic cytokine IL-11 was also induced (Fig. 2d–f), as was recently observed in MPXV-infected human skin organoids^28^. RT-qPCR confirmed MPXV-induced expression of a subset of these cytokines (*IL6*, *IL11*, *CXCL1*, and *CXCL8*) (Fig. 2g). Elevated inflammatory markers, including TNFα, IL-1β, IL-6 and IL-8, have also been observed in the blood of MPXV-infected individuals^29^. Interestingly, induction of this inflammatory response was unique to MPXV and was not induced or was poorly induced by other OPXV, including VACV and CPXV, which readily infect humans but rarely establish systemic infection in healthy individuals (Fig. 2g). MPXV infection also induced significant downregulation of gene expression and some significantly downregulated pathways were common to fibroblasts and THP-1 cells, including cell cycle, DNA replication, base excision repair and lysosome (Fig. 2h, i). Notably, enrichment analysis (KEGG) did not identify any significantly upregulated pathways related to IFN or antiviral immune defences for either MPXV strain in either cell type (Fig. 2c and Supplementary Fig. 5c–e), in agreement with the significant capacity of these viruses to suppress innate immunity (Supplementary Fig. 4).

### MPXV Clade IIb lineage B, but not lineage A, induces IFNβ expression in fibroblasts

**Fig. 3:**
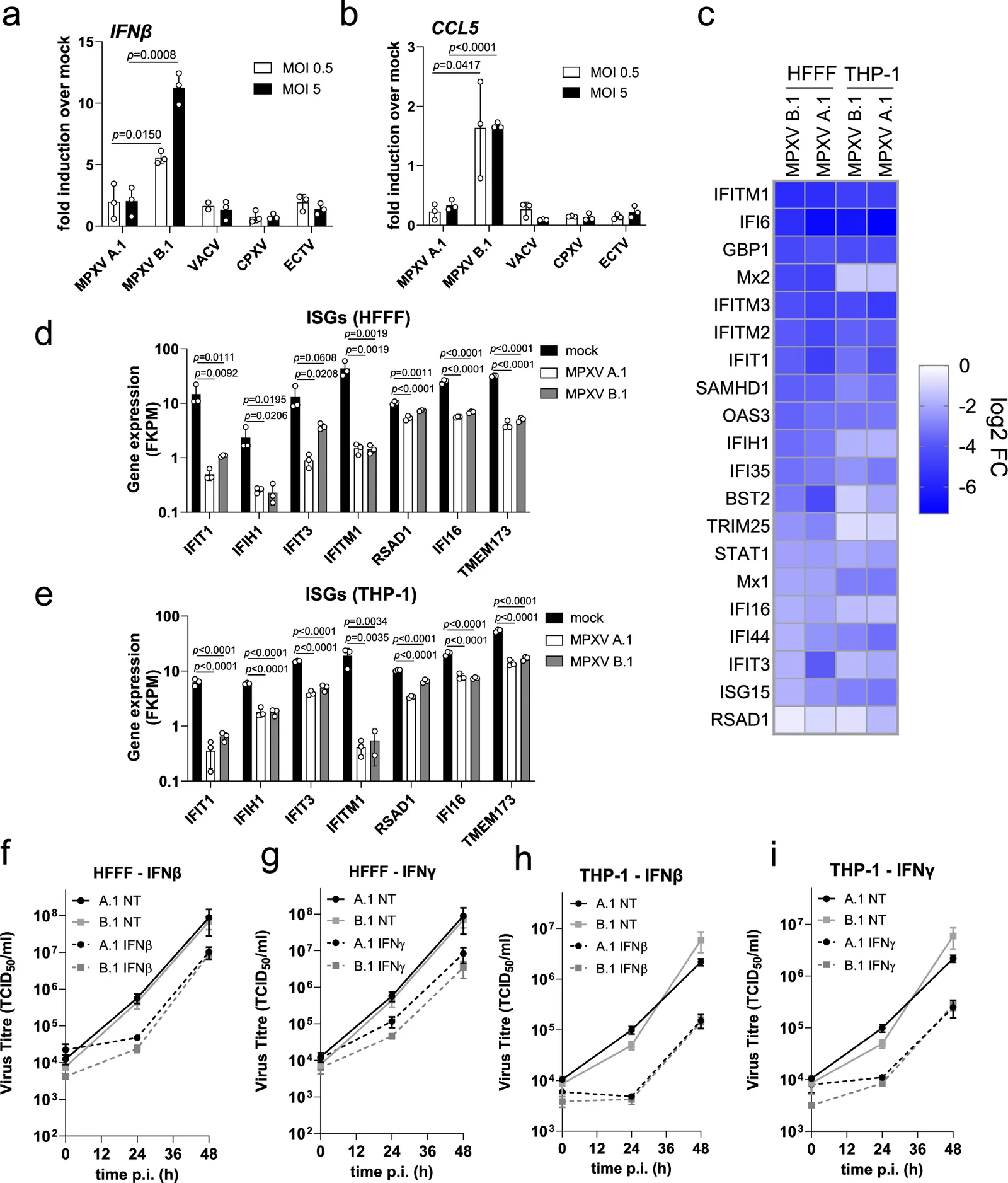
MPXV lineage B.1, but not A.1, induces IFNβ expression in fibroblasts. **a**, **b** RT-qPCR for *IFN*β (**a**) and *CCL*5 (**b**) 24 hpi from human foetal foreskin fibroblasts (HFFF) infected with 0.5 or 5 ﻿p.f.u. per cell of the indicated viruses. Data are mean ± SD. *n* = 3 biological replicates per condition. Statistical analyses were performed using an unpaired two-sided Student’s *t*-test. **c** Heat map showing log2 fold change (FC) of selected ISGs from RNAseq data of HFFF or THP-1-derived macrophages infected for 24 h with﻿ 5 p.f.u. per cell of MPXV A.1 or MPXV B.1. **d**, **e** Expression level (FKPM) of a panel of IFN-stimulated genes (ISGs) from c in HFFF (**d**) or THP-1-derived macrophages (**d**). Data are mean ± SD. *n* = 3 biological replicates per condition. Statistical analyses were performed using an ordinary one-way ANOVA with Tukey’s multiple comparisons test. **f**–**i** Viral titres at the indicated times post-infection from HFFF (**g**, **h**) or THP-1-derived macrophages (**h**, **i**) pre-treated for 16 h with 5 ng/ml of IFNβ (**f**, **h**) or IFNγ (**g**, **i**) or left non-treated (NT) and then infected with the indicated viruses at 0.05 ﻿p.f.u. per cell. All data are mean ± SD, *n* = 3 biological replicates per condition. N.b. NT datasets in (**f**–**i**) are from the same dataset presented in Fig. 1a, c. Data presented are representative of 3 (**a**, **b**) or 2 (**f**–**i**) experimental repeats, except (**c**–**e**) that are from a single RNA-seq experiment. Source data are provided as a Source Data file. MPXV monkeypox virus, VACV vaccinia virus, CPXV cowpox virus, ECTV ectromelia virus.

Although KEGG pathway analysis did not show upregulation of IFN/antiviral defences, MPXV infection did induce the expression of two type I IFNs; β and ε, in primary fibroblasts. Expression of IFNβ, however, was unique to the MPXV lineage B.1 strain and was not induced by the lineage A.1 strain (Fig. 2d). A similar result was also observed for the inflammatory chemokine *CCL5*, which was also only induced by MPXV B.1 infection in fibroblasts (Fig. 2d). Independent RT-qPCR experiments confirmed induction of these cytokines by MPXV B.1 and no, or very little, induction by MPXV A.1, or indeed other OPXV species, at multiple MOIs (Fig. 3a, b and Supplementary Fig. 7a). Type I IFN expression was not however accompanied by global IFN-stimulated gene (ISG) induction, even at very low MOI (MOI 0.05, Supplementary Fig. 7a), and in fact both MPXV lineages greatly suppressed basal ISG expression in both cell types (Fig. 3c–e). Inhibition of ISG expression despite IFNβ induction by MPXV B.1, even at very low MOI when only a small percentage of cells is infected, is likely due to the expression of the poxvirus soluble IFNα/β receptor encoded by OPG204 (VACV *B19R*). Indeed, supernatant from MPXV A.1- and B.1-, but not mock-infected cells, prevented STAT phosphorylation induced by exogenous IFNβ in the presence of a translation inhibitor (cycloheximide) to prevent viral protein expression (Supplementary Fig. 7b). In addition to blocking ISG induction, viruses are known to counteract IFN restriction by antagonising specific ISGs that are restriction factors^30^. To assess the ability of MPXV to overcome restriction by human ISGs, we measured viral replication in HFFFs and THP-1-derived macrophages that had been pre-treated with type I (IFNβ) or type II (IFNγ) IFN to upregulate ISG expression prior to infection. A dose of 5 ng/ml IFNβ (equivalent to ≥50 U/ml) and IFNγ (equivalent to ≥100 U/ml) was found to be sufficient to induce maximal expression of a subset of ISGs (CXCL10, MxA, 2′5′OAS) in both cell types (Supplementary Fig. 8). Both MPXV lineages replicated in IFN-treated human cells, reaching viral titres 1–1.5 log lower than in untreated cells (Fig. 3f–i).

### Differential innate immune control by Clade IIb lineage A and B MPXV

**Fig. 4:**
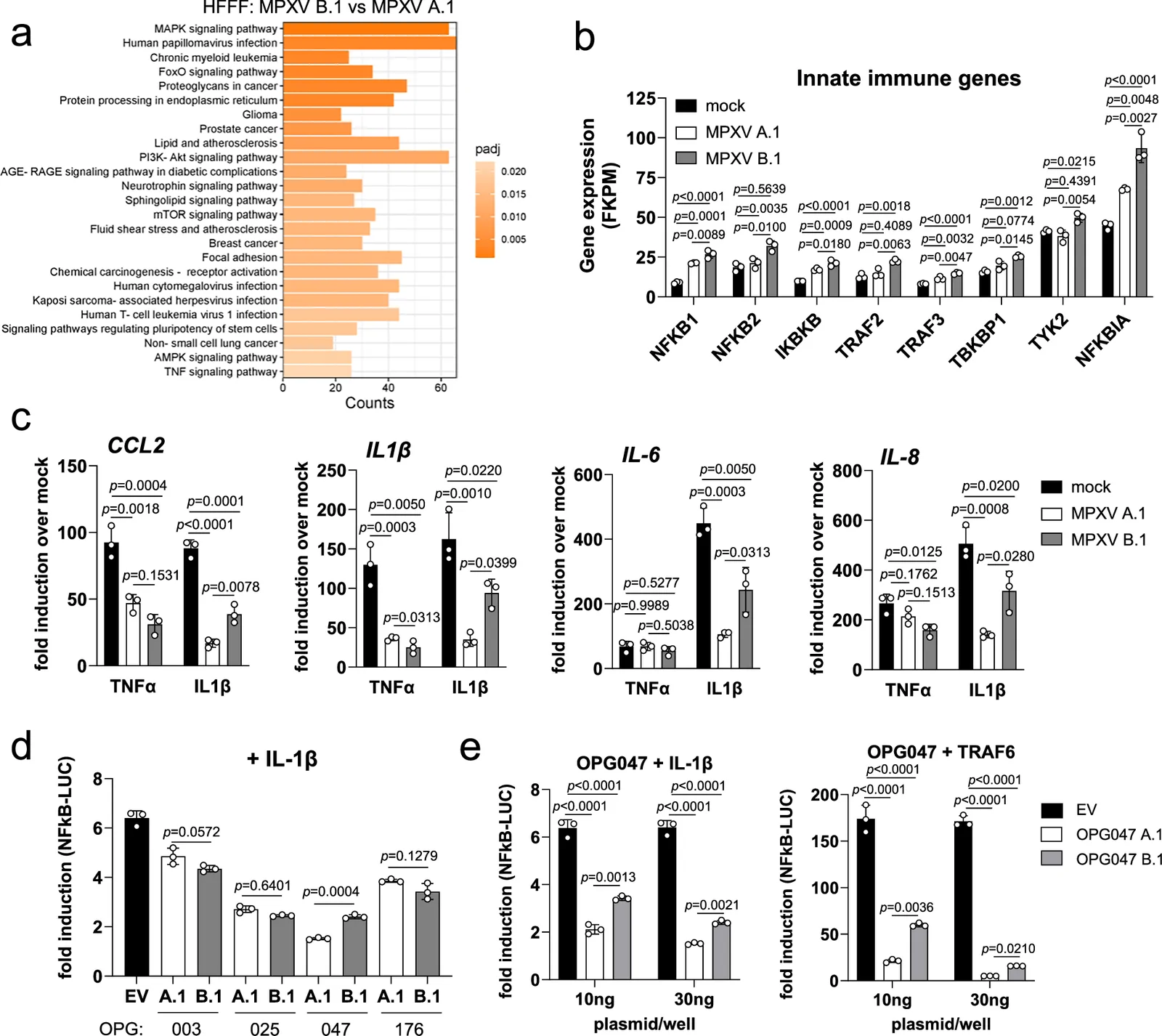
Differential innate immune control by MPXV lineages B.1 and A.1. **a** Top 25 differentially regulated pathways (KEGG analysis) from RNAseq data of human foetal foreskin fibroblasts (HFFF) infected with 5 ﻿p.f.u. per cell of monkeypox virus (MPXV) B.1 for 24 h compared with MPXV A.1-infected cells. *P* value determined by DESeq2. **b** Expression level (FKPM) of a panel of innate immune-related genes from infected THP-1-derived macrophages. Data are mean ± SD. *n* = 3 biological replicates per condition. Statistical analyses were performed using an ordinary one-way ANOVA with Tukey’s multiple comparisons test. **c** RT-qPCR for NF-κB-dependent genes from HFFF cells infected for 4 h with 5 ﻿p.f.u. per cell of the indicated viruses and then stimulated for 4 h with either TNFα (50 ng/ml) or IL-1β (10 ng/ml). Data are mean ± SD. *n* = 3 biological replicates per condition. Statistical analyses were performed using an ordinary one-way ANOVA with Tukey’s multiple comparisons test. **d** NF-κB reporter activity from HEK293T cells transfected for 24 h with 30 ng plasmid expressing the lineage A.1 or B.1 variant of the indicated MPXV orthopoxvirus genes (OPGs) or an empty vector (EV) control per well, and then stimulated for 8 h with IL-1β (25 ng/ml). *n* = 3 biological replicates per condition. Data are mean ± SD. Statistical analyses were performed using an ordinary one-way ANOVA with Tukey’s multiple comparisons test. **e** NFκB reporter activity from HEK293T cells transfected for 24 h with either 10 or 30 ng plasmid expressing the lineage A.1 or B.1 variant of MPXV OPG 047 or an empty vector (EV) control per well, and either stimulated for 8 h with IL-1β (25 ng/ml) or by co-transfection with 10 ng plasmid expressing TRAF6. *n* = 3 biological replicates per condition. Data are mean ± SD. Statistical analyses were performed using an ordinary one-way ANOVA with Tukey’s multiple comparisons test. Data presented are representative of 3 experimental repeats, except (**a**) and (**b**) that are from a single RNA-seq experiment. Source data are provided as a Source Data file.

Having observed differences in IFNβ and CCL5 expression in response to infection by lineage A and B MPXV strains, we looked for further differences in host responses to these two viruses. We therefore compared our RNA seq datasets from the two strains and identified a number of DEGs (>log2FC 0 and *P* < 0.05) in both cell types (Supplementary Fig. 9a, b). Mapping of viral transcripts showed overlapping gene expression patterns for MPXV B.1 and A.1 indicating comparable infection levels (Supplementary Fig. 9c). KEGG pathways differentially regulated by the two strains included various virus infection and innate signalling pathways such as RIG-I-like receptor and TNF signalling (Fig. 4a and Supplementary Fig. 9d). Many of the DEGs within these pathways were innate immune signalling molecules and their expression was more greatly induced by MPXV B.1 than A.1 (Fig. 4b). Many of these genes are regulated by the NF-κB family of transcription factors. Poxviruses are well known for their ability to counteract host NF-κB pathways through the expression of a battery of inhibitory proteins^27,31^. We therefore questioned whether the lineage A and B MPXV strains had differential abilities to suppress NF-κB activation and found that whilst both viruses equally modulated TNFα-induced NF-κB-dependent gene expression, the lineage B strain had a reduced capacity to suppress NF-κB-dependent gene expression downstream of IL-1β stimulation (Fig. 4c). Equivalent infection levels in these experiments were confirmed by measuring representative early (OPG035), intermediate (OPG057) and late (OPG153) viral transcripts (Supplementary Fig. 9e). To map the change(s) responsible for these differences, we cloned A.1 and B.1 versions of intracellular OPGs carrying coding changes located at the termini of the genome (regions rich in viral immunomodulators) and screened their comparative ability to suppress IL-1β-induced NF-κB activation by reporter gene assay. Of the four OPGs tested, the B.1 version of OPG047 (R48C, Supplementary Table 1) had a reduced capacity to suppress NF-κB reporter activation compared to the A.1 allele, despite equivalent protein expression (Fig. 4d and Supplementary Fig. 10). This difference was confirmed at multiple plasmid doses and downstream of TRAF6-induced NF-κB activation (Fig. 4e). OPG047 (VACV *F3L*) encodes a BTB-Kelch protein previously described to inhibit NF-κB by preventing p65 nuclear translocation through an unknown mechanism and contributes to virulence^32,33^. Like the coding change in OPG057, amino acid substitution R48C was the consequence of an APOBEC3 nucleotide mutation in B.1 (Supplementary Table 1). Taken together, these data reveal differential modulation of host responses by the 2022 lineage B outbreak MPXV relative to its West African Clade IIb ancestor, which (at least partially) was driven by APOBEC3 editing.

### Reduced virulence of the 2022 lineage B MPXV in vivo

**Fig. 5:**
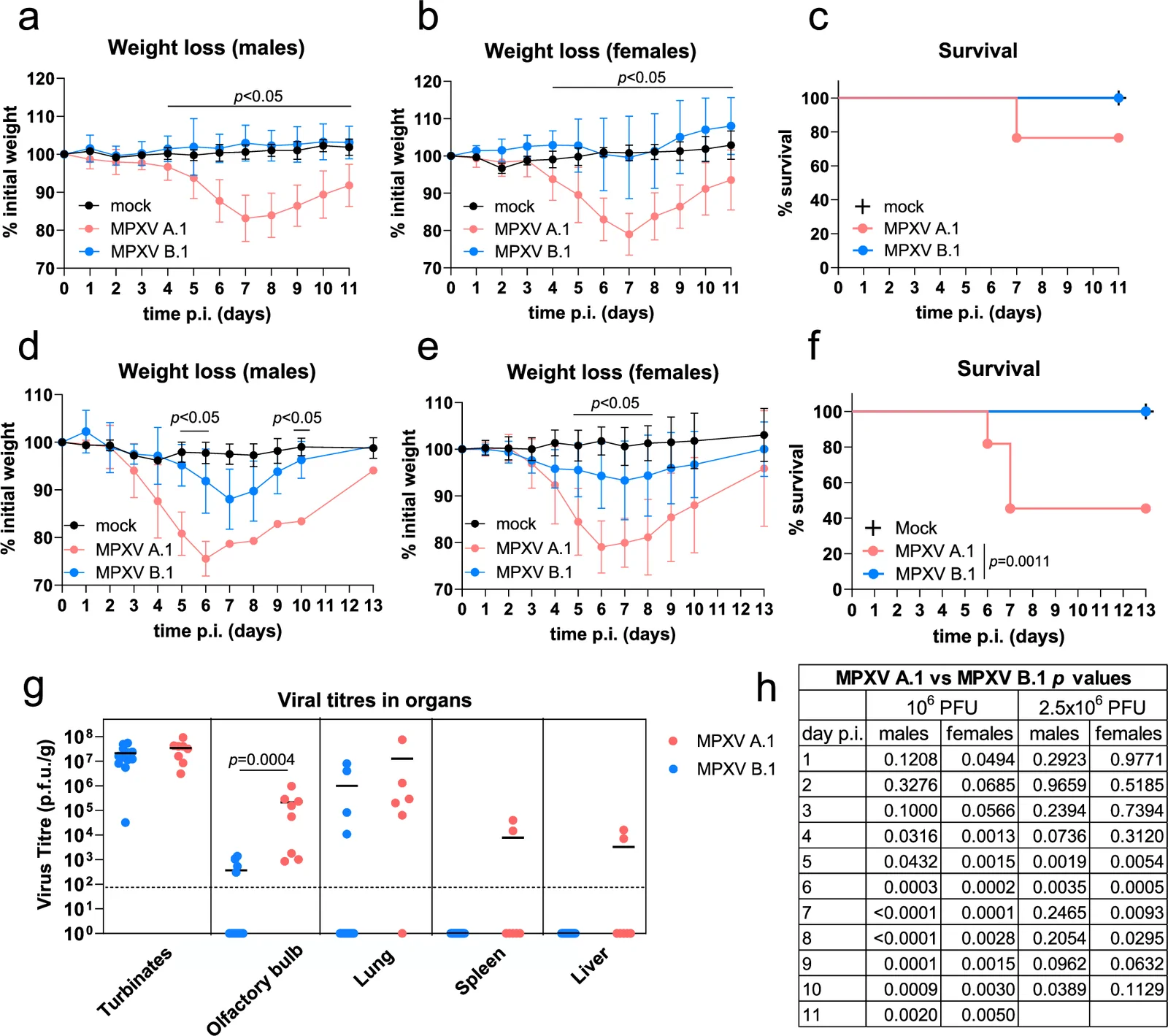
Reduced virulence of MPXV lineage B.1 relative to A.1 in vivo. **a**, **b** Groups of male (**a**, *n* = 3 mock, *n* = 7 monkeypox virus (MPXV) A.1, *n* = 6 MPXV B.1) and female (**b**, *n* = 3 mock, *n* = 10 MPXV A.1, *n* = 8 MPXV B.1) CAST mice were infected intranasally with 10^6^ p.f.u. of the indicated viruses. Mice were monitored daily for bodyweight and survival. Weight data are expressed as the mean ± SD of the animals’ weights compared to their original weight at the day of inoculation. Statistical analyses were performed using a multiple un-paired *t*-test with a 1% false discovery rate and days at which significant differences (*p* < 0.05) were found between MPXV A.1 and MPXV B.1 are indicated with the solid line. All *p* values are shown in (**h**). **c** Survival data of males and females combined from (**a**, **b**). **d**, **e** Groups of male (**d**, *n* = 3 mock, *n* = 4 MPXV A.1, *n* = 5 MPXV B.1) and female (**e**, *n* = 4 mock, *n* = 7 MPXV A.1, *n* = 8 MPXV B.1) CAST mice were infected intranasally with 2.5 × 10^6^ p.f.u. of the indicated viruses and monitored as above. Weight data are presented and analysed (multiple un-paired *t*-tests with a 1% false discovery rate) also as above. **f** Survival data of males and females combined from (**c**, **d**). Statistical analyses were performed using a Mantel–Cox test. **g** Viral titres in organs from CAST mice (*n* = 8 MPXV A.1, *n* = 12 MPXV B.1) 5 days post intranasal infection with 2.5 × 10^6^ p.f.u. of the indicated viruses. Solid horizontal lines represent the mean titre. Dotted horizontal line indicates limit of detection. Statistical analyses were performed using a two-sided Mann–Whitney test. **h** Statistical analyses summary from (**a**, **b**, **d**, and **e**). Data represented are from in vivo experiments each performed once. Source data are provided as a Source Data file.

A reduced ability to disseminate^34^ or to counteract host innate immune responses^25,35^, as observed here for the 2022 lineage B strain relative to its lineage A.1 ancestor, is associated with viral attenuation in small rodent infection models for other OPXV. To assess virulence of these lineages, we infected groups of male and female CAST mice intranasally (i.n.) with either 10^6^ (Fig. 5a–c) or 2.5 × 10^6^ (Fig. 5d–f) plaque-forming units (p.f.u.) of each MPXV lineage and monitored animals daily for survival and weight loss. The virus preparations used were grown in the same cell type in parallel, semi-purified in the same way and shown to have the same protein to infectivity ratios, so that any differences seen were likely due to biological differences in the viruses rather than differences in the purity of virus preparations. Under these conditions, infection with lineage A.1 MPXV induced more severe disease than lineage B.1. On average at the higher viral dose, mice infected with A.1 lost 23% of their body weight 7 days after infection (Fig. 5d, e), reaching experimental humane endpoints and resulting in a 54% mortality rate (6/11) (Fig. 5f). On the contrary, lineage B.1 MPXV induced 7% weight loss (Fig. 5d, e) and failed to cause mortality (Fig. 5f). Furthermore, both lineages exhibited similar replication at the inoculation site (nasal turbinates, Fig. 5g). However, viral titres in the distant organs such as the olfactory bulb were significantly lower in MPXV B.1-infected mice indicating a defect in viral dissemination (Fig. 5g). Attenuation of the clade IIb MPXV lineage B relative to zoonotic clade IIa and clade I viruses has previously been shown in CAST mice^22^. Our data now reveal virulence differences within clade IIb MPXV, where lineage B.1 is attenuated relative to a closely related lineage A.1 isolate.

## Discussion

Between September 2017 and September 2018, an outbreak of MPXV clade IIb (now termed lineage A.1) involving 122 confirmed or probable cases was declared in Nigeria^10^. MPXV genomes recovered from Nigeria between the time of the Nigerian outbreak and the 2022 global outbreak revealed an unusual diversification characterised by the accumulation of mutations consistent with APOBEC3 activity^2^. Lineage A has established sustained human-to-human transmission and at present, descendant sub-lineages co-circulate endemically^36^. Although its origin remains obscure, the lineage B descends from the same zoonotic event that caused the Nigerian outbreak and can be phylogenetically traced back to lineage A.1^1–3^. The APOBEC3 family has expanded and diverged into 7 paralogues in humans^37^, whereas only one gene exists in rodents, the major OPXV reservoir. Hence, the elevated deamination resulting in the lineage B.1 mutational footprint is consistent with viral exposure to the more complex APOBEC3 system in humans relative to rodents. Certainly, 23/24 amino acid substitutions in 2022 global B.1 MPXV relative to ancestral lineages from 2018 reported here are compatible with APOBEC3 deamination (Supplementary Table 1). At present the distribution of these mutations in MPXV genomes suggest they are random and their presence serves as a marker of human-to-human transmission^2,3^. Our data indicate that, in addition, the mutational footprint in the lineage B MPXV results in both differential spread and host responses, including increased expression of innate immune-related genes such as IFNβ, diminished capacity to counteract host NF-κB responses, and consistent with this, reduced virulence in mice relative to lineage A.1. Genetic screening of intracellular OPG alleles located in terminal regions identified amino acid substitution R48C in OPG047 as responsible for the defect in innate immune control, whereas recombinant viruses carrying the mutated lineage B.1 allele for OPG057 showed a defect in spread. Both OPG047 R48C and OPG057 E353K mutations are compatible with the action of APOBEC3. Hence, despite the large 186,000 bp MPXV genome, APOBEC3-like activity has the capacity to drive non-neutral changes in MPXV that result in novel phenotypes.

Our data show that infection with the globally circulating MPXV lineage B.1 results in IFNβ induction and diminished capacity to suppress host innate responses relative to lineage A, which at present remains endemic to West Africa. Induction of IFNβ did not result in ISG upregulation in infected cells, presumably due to the plethora of immune antagonists encoded by OPXV, including the action of the soluble IFNα/β decoy receptor, but would be expected to modulate the immune response in vivo through paracrine activity on uninfected cells and tissues, establishing an antiviral state. In addition, we observed reduced capacity for B.1 to disseminate in vitro and to cause disease in CAST mice. In humans, clinical data report 60% MPXV-infected patients in Nigeria (where lineage A dominates) exhibited substantial viral dissemination with >100 lesions^38^, whereas > 64% of patients infected with the derivative lineage B.1 MPXV showed fewer than 10 lesions, all localised to the anogenital area^12,39^. Higher lesion count is known to correlate with mpox disease severity^40^. Such reduced dissemination and disease severity for lineage B.1 in humans is consistent with reduced extracellular virus, poorer innate immune control and attenuation in virulence in an animal model. More recently, an mpox outbreak in Sierra Leone has recorded >4500 confirmed mpox cases and a CFR of 0.7%^13^, significantly higher than the CFR for global mpox (~0.2%) and the novel Clade Ib variant that has emerged in DRC (~ 0.3%)^17,18^. The Sierra Leone mpox outbreak derives from Nigerian Clade IIb lineage A.2, which does not contain the APOBEC3 attenuating mutations identified here in B.1. Hence, the morbidity and mortality of future Clade IIb lineages might be higher than that expected based on the 2022 lineage B.1 outbreak. Whether APOBEC3 is also inducing attenuation in Clade Ib is the subject of ongoing work.

A process of attenuation by loss-of-function is thought to have occurred after the intentional release and subsequent evolution of myxoma virus (a poxvirus of leporids) in Australia in the 1950s to control the introduced European rabbits^41^. Most of these now dominant myxoma virus strains are attenuated and unable to counteract European rabbit PKR due to a single amino acid substitution in one of the viral PKR antagonists^42^. It is thought that loss of immunosuppressive capacity by gene loss also occurred during VARV evolution^43^. Our data show that APOBEC3 editing is a mechanism of MPXV phenotypic variation in humans that attenuated the MPXV strain that caused the 2022 mpox global outbreak. Whether APOBEC3-driven attenuation impacted transmission and global dissemination of mpox, however, remains to be determined and other, non-biological factors are likely to have contributed^44,45^. It is important to note, for example, that EEV are important for long-range spread within the host, but not for host-to-host transmission, which is mediated by IMV^46^. Hence, attenuation in EEV titre and spread is expected to influence disease severity but may have limited impact on transmission. Mpox transmission via skin primary rash, as seen since 2017, leads to compressed transmission chains relative to historical transmission routes by respiratory droplet or fomites from secondary disseminated rash^45^. In a global setting, this could lead to transmission chains being further compressed relative to endemic settings, generating mutations in lineage B.1 faster than lineage A. Consequently, the constant increase of APOBEC3-like mutations is not itself evidence of MPXV adaptation to humans. However, this mutational drift provides opportunities for selection and may favour the appearance of novel variants. The lineage B.1 viruses used in this study were collected in the UK and Spain in June 2022, shortly after the emergence of MPXV in non-endemic countries in May 2022, suggesting that this lineage had acquired the attenuated phenotypes reported here prior to, or early in, the global outbreak, and descendant lineages are likely to have them as well. The ability of host enzymes, together with other reported mechanisms^47^, to generate novel phenotypic profiles is therefore an important consideration for surveillance and control of current and future MPXV epidemics.

## Methods

### Cells and reagents

BSC-40 (ATCC #CRL-2761), HFFF2 (ECACC #86031405), U2OS (ATCC #HTB-96) and HEK293T (ATCC #CRL-3216) cells were cultured in Dulbecco modified Eagle medium (Sigma) and THP-1 cells (THP-1 IFIT-1 Gluc cells^48^) were cultured in RPMI medium (Gibco), all supplemented with 10% foetal bovine serum (FBS, Biowest) and 100 U/mL penicillin and 100 µg/mL streptomycin (Pen/Strep; Life Technologies). THP-1 cells were differentiated for 48 h in the presence of 50 ng/mL PMA (Peprotech). HT-DNA (Sigma), 2′3′-cGAMP (Invivogen) and polyI:C (Invivogen) were used to stimulate cells by lipofectamine 2000 (Invitrogen) transfection according to the manufacturer’s instructions. Human IFNβ and γ were obtained from Peprotech and dissolved in water at 100 µg/ml (equivalent to ≥10^6^ U/mL for IFNβ and ≥2 × 10^6^ U/mL for IFNγ). Human TNFα and IL-1β were also from Peprotech. Universal type I IFN was from PBL Assay Science. JAK inhibitor ruxolitinib and AP-1 inhibitor SR11302 were purchased from Cambridge Bioscience. Cycloheximide was purchased from Sigma.

### Virus stocks and cell infections

MPXV Clade IIb lineage B viruses (MPXV_CVR_S1; accession number ON808413 and MPXV_CBM; accession number GCA_964276875) were isolated in 2022 from patients at the University of Glasgow-Centre for Virus Research (CVR) and at the Centro de Biologia Molecular Severo Ochoa (CBM) in Madrid, Spain, respectively, and differ in 1 coding change. Lineage A viruses (MPXV_UK_P2; accession number MT903344 and MPXV_UK_P3; accession number MT903345) were isolated in 2018 at the UK Health Security Agency and are genetically identical. Viruses were amplified in BSC-40 (MPXV_CVR_S1 and MPXV_UK_P3) and BSC-1 (MPXV_CBM and MPXV_UK_P2) cells before purification and titration by plaque assay. Both MPXV_CVR_S1 and MPXV_CBM isolates were characterised for viral growth with indistinguishable results between them and relative to their MPXV_UK_P2 and MPXV_UK_P3 lineage A comparators. Other viruses used were VACV Western Reserve (AY243312), CPXV Brighton-Red (AF482758.2), and ECTV Moscow (AF012825). ECTVΔvSlfn has been described in ref. ^25^. VACV, CPXV and ECTV were expanded in BSC-40 cells and purified before titration by plaque assay^49^. Purification was performed by ultracentrifugation of cytoplasmic extracts over a 36% (w/v) sucrose cushion (32,900 × *g* for 80 min at 4 °C) prior to use. Protein-to-infectivity ratios were calculated for all MPXV isolates and shown to be indistinguishable (mean ± SD: 1.09 ± 0.2 × 10^9^ PFU/mg for A.1, 1.22 ± 0.55 × 10^9^ PFU/mg for B.1). All infections were performed in medium supplemented with 2.5% FBS. Cells were initially infected in a minimal volume of medium on ice for 60–90 min before unbound virus was washed off with medium and cells transferred to 37 °C. All infection experiments were performed in a Biosafety level 3 laboratory at The Pirbright Institute, Surrey. Plaque size was measured using ImageJ. For MPXV cell associated and extracellular virus growth curves, virus titre was quantified by tissue culture infectious dose 50 (TCID50) assay according to the Reed and Muench method^49,50^. TCID50 assays were performed on BSC-40 cells and incubated for 6–7 days. For VACV, virus titres from growth curves were calculated by plaque assay on BSC-40 cells, incubated for 3 days.

### Comet tail assays

BSC-40 or U2OS cells were seeded in 6-well plates (for MPXV) or 10 cm dishes (for VACV) and infected the following day. Cells were initially infected in a minimal volume of medium (supplemented with 2.5% FBS) with threefold dilutions of virus (aiming 30 plaques per well) for 60–90 min before unbound virus was washed off, fresh medium was added (liquid overlay) and cells transferred to 37° C. Cells were incubated for 3 days for VACV and 5 days for MPXV before staining with crystal violet. For experiments with ruxolitinib, this was added after the initial 60–90 min adsorption period. Plaque area and comet tail length were measured using ImageJ.

### Construction of VACV F13 E353K

For construction of a VACV carrying WT F13 or F13 with an E353K mutation from its natural locus, plasmids containing either the WT coding sequence of VACV F13 (pF13WT) or with an E353K mutation (pF13E353K), flanked by 200-bp of the 5′ and 3′ regions of the *F13L* locus, and the Escherichia coli guanylphosphoribosyl transferase (Ecogpt) gene fused in-frame with the enhanced green fluorescent protein (EGFP) gene were generated^35^. HEK293T cells were infected with a VACV strain Western Reserve lacking *F13L*^51^ and then transfected with either pF13WT or pF13 E353K. EGFP-positive plaques were then selected on BSC-40 cells as described previously^52^. Viral genotypes were confirmed by sequencing following lysis and proteinase K treatment of infected BSC-40 cells and PCR using primers that anneal to the flanking regions of *F13L*.

### IFN sensitivity assays

For IFN sensitivity assays cells were seeded at 2.5 × 10^5^ cells/well in 24-well plates. The following day cells were treated overnight with 5 ng/mL IFNβ or γ, or mock-treated with medium and then infected the next morning on ice at an MOI 0.05 in 200 μL/well medium. After 60 min the inoculum was removed and the cells washed once with medium to remove unbound virus before 500 μL medium was added per well and the cells transferred to 37 °C. Virus was harvested at 0, 24, and 48 hpi by scraping the cells in their medium and freeze-thawing three times before calculation of viral titres by TCID50 assay according to the Reed and Muench method^50^. TCID50 assays were performed on BSC-40 cells and incubated for 6–7 days.

### RT-qPCR

For qPCR cells were seeded at 2.5 × 10^5^ cells/well in 24-well plates and harvested in 140 µL PBS + 560 µL buffer AVL (QIAgen) containing 1 % (v/v) triton X-100. After incubation at room temperature for 10 min, 560 µL ethanol was added and incubated for a further 20 min before extracting total RNA using the RNeasy mini RNA extraction kit (QIAgen), including on-column DNase treatment according to the manufacturer’s instructions. Five hundred ng RNA was used to synthesise cDNA using Superscript III reverse transcriptase (Invitrogen), also according to the manufacturer’s protocol. cDNA was diluted 1:5 in water and 1.5 μL was used as a template for real-time PCR in a 5 μL reaction volume total containing SYBR® Green PCR master mix (Applied Biosystems). Gene expression was quantified on a Quant Studio 5 real-time PCR machine (Applied Biosystems) with Quantstudio design and analysis software (version 1.5.2). Expression of each gene was normalised to an internal control (*18S RNA*) and these values were then normalised to mock-treated control cells to yield a fold induction. The following primers were used to detect host genes:

18S RNA: Fwd 5′-GTAACCCGTTGAACCCCA-3′, Rev 5′-CCATCCAATCGGTAGTAGCG-3′

CXCL-10: Fwd 5′-TGGCATTCAAGGAGTACCTC-3′, Rev 5′-TTGTAGCAATGATCTCAACACG-3′

MxA: Fwd 5′-CCCCAGTAATGTGGACATCG-3′, Rev 5′-ACCTTGTCTTCAGTTCCTTTGT-3′

2′5′ OAS: Fwd 5′-TGTGTGTGTCCAAGGTGGTA-3′, Rev 5′-TGATCCTGAAAAGTGGTGAGAG-3′

CXCL1: Fwd 5′-CTGCGCTGCCAGTGCTTGCA-3′, Rev 5′-TGTGGCTATGACTTCGGTTTG-3′

CXCL8 (IL-8): Fwd 5′- AGAAACCACCGGAAGGAACCATCT-3′, Rev 5′- AGAGCTGCAGAAATCAGGAGGGCT-3′

IL6: Fwd 5′-AAATTCGGTACATCCTCGACG-3′, Rev 5′-GGAAGGTTCAGGTTGTTTTCT-3′

IL11: Fwd 5′-GGACATGAACTGTGTTTGCC-3′, Rev 5′-CCGTCAGCTGGGAATTTGTC-3′

IL1β: Fwd 5′-ACAGATGAAGTGCTCCTTCCA-3′, Rev 5′-GTCGGAGATTCGTAGCTGGAT-3′

ICAM1: Fwd 5′-TCTGTGTCCCCCTCAAAAGTC-3′, Rev 5′-GGGGTCTCTATGCCCAACAA-3′

IFIT1: Fwd 5′-CCTGAAAGGCCAGAATGAGG-3′, Rev 5′-TCCACCTTGTCCAGGTAAGT-3′

IFNβ: Fwd 5′-ACATCCCTGAGGAGATTAAGCA-3′, Rev 5′-GCCAGGAGGTTCTCAACAATAG-3′

CCL5: Fwd 5′-CCCAGCAGTCGTCTTTGTCA-3′, Rev 5′-TCCCGAACCCATTTCTTCTCT-3′

HSPA6: Fwd 5′-GATGTGTCGGTTCTCTCCATTG-3′, Rev 5′-CTTCCATGAAGTGGTTCACGA-3′

HSPA1A: Fwd 5′-AGCAGGTGTGTAACCCCATC-3′, Rev 5′-GCAGCAAAGTCCTTGAGTCC-3′

DUSP1: Fwd: 5′-GCTCAGCCTTCCCCTGAGTA-3′, Rev 5′-GATACGCACTGCCCAGGTACA-3′

CCL2: Fwd 5′-CAGCCAGATGCAATCAATGCC-3′, Rev 5′-TGGAATCCTGAACCCACTTCT-3′

Primers to detect viral genes were as follows

OPG035: Fwd 5′-ATCATCCTAAAGAGTATGGTG-3′, Rev 5′-GCTAGATAGATTGAATCAACC-3′

OPG057: Fwd 5′-CGATTTACTGTGGCTAGATAC-3′, Rev 5′-ATATCACTTCGGCAAATTTCG-3′

OPG153: Fwd 5′-ATCTCCCATGTGGTGGAATAC-3′, Rev 5′-GTTGATAGGTTAGAACATCAC-3′

### Transcriptomics and bioinformatics

For transcriptomics experiments cells were seeded at 2.5 × 10^5^ cells/well in 24-well plates and infected in triplicate with MPXV A.1, MPXV B.1 or mock-infected as a control at an MOI 5 for 24 h. RNA was purified as described above using the RNeasy mini RNA extraction kit with on-column DNase treatment and sent for bulk RNA sequencing by Novogene. Messenger RNA was purified from total RNA using poly-T oligo-attached magnetic beads. After fragmentation, the first strand cDNA was synthesised using random hexamer primers, followed by the second strand cDNA synthesis using either dUTP for directional library or dTTP for non-directional library. The library was checked with Qubit and real-time PCR for quantification and bioanalyzer for size distribution detection. Quantified libraries were then pooled and sequenced on an Illumina platform. RNA-seq reads were aligned to the *Homo Sapiens* genome using HISAT2^53^ and featureCounts v1.5.0-p3 was used to count the read numbers mapped to each gene and the FPKM of each gene was calculated based on the length of the gene and reads count mapped to it. DEG were calculated using DESeq2^54^. The resulting *P*-values from DESeq2 were adjusted using the Benjamini and Hochberg’s approach for controlling the false discovery rate. Genes with an adjusted *P*-value < 0.05 found by DESeq2 were assigned as differentially expressed. DEGs were then used for KEGG pathway enrichment analysis^55^. Pathway *p*-values < 0.05 were considered significant. Heat maps were generated using GraphPad Prism (version 10) and KEGG pathway analysis figures were generated using SRPlot (accessed from https://www.bioinformatics.com.cn/srplot). For mapping of viral gene reads, RNA sequencing reads were aligned to a MPXV reference genome (NC_063383.1) using the HISAT2 software. Following alignment, the featureCounts tool was employed to quantify the read counts mapped to each gene. These counts were then normalised to counts per million to facilitate comparison across samples.

### Reporter gene assays

Dual luciferase reporter gene assays in HEK293T cells were performed as we have described previously^25,56^. Luminescence measurements were made on a CLARIOstar plate reader (BMG Labtech) with CLARIOstar software (version 5.20 RS). Plasmids encoding MPXV Clade IIb lineage B.1 OPGs 003, 025, 047, and 176 (obtained from Mike Weekes, University of Cambridge) were cloned into a modified pcDNA4 vector with 3 copies of the FLAG tag at the N terminus^57^. Lineage A.1 versions of these genes were generated by site-directed mutagenesis using Pfu Turbo DNA polymerase (Agilent) using the following primers:

OPG003_N264D_Fwd: 5′ TACCAGGATCAGAAATAATCTTGATTGTACACCC 3′

OPG003_N264D_Rev: 5′ TACTCCATGATGGGTGTACAATCAAGATTATTTC 3′

OPG025_D423A_Fwd: 5′ TGTATCGAGTCTCACAGTGTTGCCCTGATCGAATG 3′

OPG025_D423A_Rev: 5′ ATCTATGAGCCATTCGATCAGGGCAACACTGTGAG 3′

OPG047_C48R_Fwd: 5′ GAAGAAGCTCTCACCATACTTTCGCACACACCTG 3′

OPG047_C48R_Rev: 5′ TGTACTTTTGGCGCAGGTGTGTGCGAAAGTATGG 3′

OPG176_Y221H_Fwd: 5′ CAGCATCGAGGACGAATTTGATCATTTCGAAGACG 3′

OPG176_Y221H_Fwd: 5′ TGGAAAGGTCATCGTCTTCGAAATGATCAAATTCG 3′

### Immunoblotting

For immunoblotting cells were seeded at 5 × 10^5^ cells/well in 12-well plates and harvested in 120 µL RIPA buffer (50 mM Tris-HCl pH8, 150 mM NaCl, 1% (v/v) NP-40, 0.5% (w/v) sodium deoxycholate, 0.1% (w/v) SDS) supplemented with protease and phosphatase and protease inhibitors (Roche) and benzonase (EMD Millipore). Samples were boiled for 5 min in boiled in Laemmli buffer (final concentration 50 mM Tris-HCl pH 6.8, 2% (w/v) SDS, 10% (v/v) glycerol, 0.1% (w/v) bromophenol blue, 100 mM betamercaptoethanol) and proteins were separated by SDS-PAGE on 4–12% bis-tris polyacrylamide gels (Invitrogen). After PAGE, proteins were transferred to a Hybond ECL membrane (Amersham Biosciences) using a semi-dry transfer system (Biorad). Primary antibodies were from the following sources: pSTING Ser366 (D7C3S, Cell Signaling Technologies 19781S, 1:1000), STING (D2P2F, Cell Signaling Technologies 13647S, 1:1000), pIRF3 Ser386 (EPR2346, Abcam AB76493, 1:1000), IRF3 (EPR2418Y, Abcam AB68481, 1:1000), pSTAT1 Tyr701 (58D6, Cell Signaling Technologies 9167S, 1:1000), STAT1 (D1K9Y, Cell Signaling Technologies 14994S, 1:1000), pSTAT2 Y690 (D3P2P, Cell Signaling Technologies 88410T, 1:1000), STAT2 (D9J7L, Cell Signaling Technologies 72604T, 1:1000), IFIT1 (D2X9Z, Cell Signaling Technologies 14769S, 1:1000), MxA (D3W7I, Cell Signaling Technologies 37849S, 1:500), tubulin (DM1A, Merck Millipore 05-829, 1:5000), actin (2D4H5, Proteintech 66009-1-Ig, 1:10,000), FLAG (M2, Sigma F1804, 1:5000), ERK (Proteintech 66192-1-IG, 1:1000), pERK T202/Y204 (Cell Signaling Technologies 9101S, 1:1000), p38 (Cell Signaling Technologies 9212S, 1:1000), pp38 T180/Y182 (Cell Signaling Technologies 9211S, 1:1000), JNK (Cell Signaling Technologies 9252S, 1:1000), pJNK T183/Y185 (Cell Signaling Technologies 9251S, 1:1000), DUSP1 (E6T5S, Cell Signaling Technologies 35217T, 1:1000), OPG057 (1:1000) and OPG079 (1:2000) (both from D. Ulaeto, Defence Science and Technology Laboratory). Primary antibodies were detected with goat-anti-mouse/rabbit IRdye 680/800 infrared dye secondary antibodies and membranes imaged using an Odyssey Infrared Imager with ImageStudio Acquisition Software (version 5.2) (LI-COR Biosciences).

### MPXV infection of mice

The experiments described and CAST/EiJ mice (originally obtained from The Jackson Laboratory, Strain #:000928) breeding were carried out at the CBM in Madrid (Spain) under pathogen-free conditions. Mice were housed in ventilated racks within biological safety level 3 (BSL‑3) containment facilities at 20–24 °C with a relative humidity of 40–60%, with ad libitum access to water and irradiated universal feed for mice and hamsters. Environmental enrichment included cylindrical tunnels, compressed cellulose squares, wooden gnawing blocks, and sunflower seeds, and animals were acclimatised to BSL‑3 conditions for one week. All experimental procedures were approved by the Ethical Review Board of CBM and CSIC and by the local authorities (Comunidad de Madrid) under reference PROEX 241.1/21, and were performed by smallpox-vaccinated investigators. Randomly assigned groups of 7–12-week-old (12–16 × *g*) male and female CAST/EiJ mice were anesthetised by isoflurane inhalation immediately prior to intranasal (i.n.) infection with sucrose‑purified viral stocks, receiving a single 10 µl dose containing 10⁶ or 2.5 × 10⁶ p.f.u. of MPXV lineage A.1 (MPXV_UK_P2) or lineage B.1 (MPXV-CBM). Following infection, viral inocula were back‑titrated by plaque assay to confirm the administered dose, and animals were monitored daily for survival, weight, temperature, and clinical signs of disease. For viral load determination in organs, infected mice were euthanised by CO₂ asphyxiation at 5 days post-infection and selected organs were aseptically collected, weighed, homogenised in 1 ml PBS, and subjected to three freeze–thaw cycles. Viral titres were then determined by plaque assay in BSC‑1 cells as previously described.

### Statistical analysis

Statistical analyses were performed using either a two-tailed unpaired Student’s *t*-test (with Welch’s correction where variances were unequal), an ordinary one-way ANOVA with multiple comparisons, multiple *t*-tests, a Mantel–Cox test or a Mann–Whitney test as indicated in the figure legends. All analyses were performed with GraphPad Prism version 10.

### Reporting summary

Further information on research design is available in the Nature Portfolio Reporting Summary linked to this article.

## Supplementary information

Supplementary Information

Reporting Summary

Transparent Peer Review file

## Source data

Source Data

## Data Availability

OPXV nucleotide sequences cited in this study are available on NCBI GenBank: MPXV_CVR_S1 (ON808413), MPXV_CBM (GCA_964276875), MPXV_UK_P2 (MT903344), MPXV_UK_P3 (MT903345), VACV Western Reserve (AY243312), CPXV Brighton-Red (AF482758.2) and ECTV Moscow (AF012825). The RNA-sequencing data generated in this study have been deposited in the Gene Expression Omnibus database under accession code GSE338258. Source data are provided with this paper.
