# Supplementary material for: APOBEC3-driven attenuation of the 2022 global outbreak monkeypox virus relative to its clade IIb ancestor
Source: Nat Commun. 2026 Aug 11;17:9630. doi: 10.1038/s41467-026-76580-7 (PMC13554265; doi:10.1038/s41467-026-76580-7)
Supplement: Supplementary file 1 [file 41467_2026_76580_MOESM1_ESM.pdf]

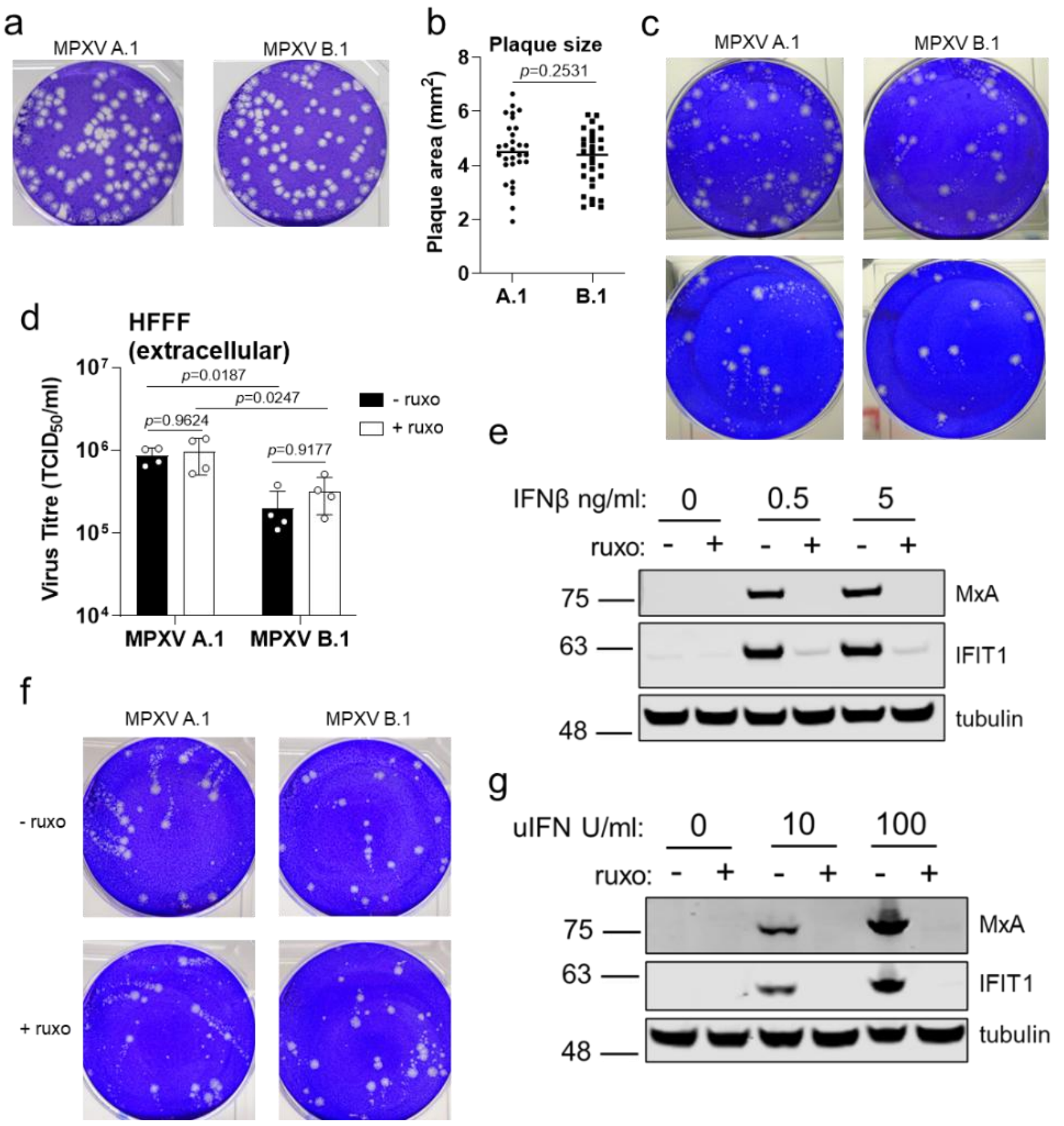

Supplementary Data Fig 1. MPXV Clade IIb lineage B.1 has a defect in viral spread.

a, Representative plaques formed by monkeypox virus (MPXV) A.1 and B.1 under a semi-solid overlay at 96 hpi on BSC-40 cells.

b, Plaque area quantification of 30 plaques from **a**. The horizontal bar represents the median. Statistical analyses were performed using an unpaired two-sided Student's t-test.

c, Representative comet tail assay from U2OS cells infected with MPXV A.1 or B.1 for 5 days with a liquid overlay.

d, MPXV A.1 and B.1 extracellular virus titre at 72 h in human foetal foreskin fibroblasts (HFFF) in the presence or absence of 2 μM ruxolitinib (ruxo). n = 4 biological replicates per condition. Data are mean ± SD. Statistical analyses were performed using an ordinary one-way ANOVA with Tukey's multiple comparisons test.

e, Immunoblot of HFFF cells treated for 16 h with the indicated concentrations of human IFNβ in the presence or absence of 2 μM ruxolitinib (ruxo).

f, Representative comet tail assay from BSC-40 cells infected with MPXV A.1 or B.1 for 96 h with a liquid overlay in the presence or absence of 1 μM ruxolitinib (ruxo).

g, Immunoblot of BSC-40 cells treated for 16 h with the indicated concentrations of universal IFN in the presence or absence of 1 μM ruxolitinib (ruxo).

All data presented are representative of at least 2 experimental repeats. Source data are provided as a Source Data file. Source data for immunoblots are provided at the end.

```
MPXV_A.1        MWPFASVPAGAKCRLVETLPENMDFRSDHLTTFECFNEIITLAKKYIYIASFCCNPLSTT
MPXV_B.1        MWPFASVPAGAKCRLVETLPENMDFRSDHLTTFECFNEIITLAKKYIYIASFCCNPLSTT
VACV            MWPFASVPAGAKCRLVETLPENMDFRSDHLTTFECFNEIITLAKKYIYIASFCCNPLSTT
                ************************************************************

MPXV_A.1        RGALIFDKLKEVSEKGIKIIVLLDERGKRNLGELQSHSPDINFITVNIDKKNNVGLLLGC
MPXV_B.1        RGALIFDKLKEVSEKGIKIIVLLDERGKRNLGELQSHSPDINFITVNIDKKNNVGLLLGC
VACV            RGALIFDKLKEASEKGIKIIVLLDERGKRNLGELQSHCPDINFITVNIDKKNNVGLLLGC
                ***********.*************************.**********************

MPXV_A.1        FWVSDDERCYVGNASFTGGSIHTIKTLGVYSDYPPLATDLRRRFDTFKAFNSAKNSWLNL
MPXV_B.1        FWVSDDERCYVGNASFTGGSIHTIKTLGVYSDYPPLATDLRRRFDTFKAFNSAKNSWLNL
VACV            FWVSDDERCYVGNASFTGGSIHTIKTLGVYSDYPPLATDLRRRFDTFKAFNSAKNSWLNL
                ************************************************************

MPXV_A.1        CSAACCLPVSTAYHIKNPIGGVFFTDSPEHLLGYSRDLDTDVVIDKLKSAKTSIDIEHLA
MPXV_B.1        CSAACCLPVSTAYHIKNPIGGVFFTDSPEHLLGYSRDLDTDVVIDKLKSAKTSIDIEHLA
VACV            CSAACCLPVSTAYHIKNPIGGVFFTDSPEHLLGYSRDLDTDVVIDKLKSAKTSIDIEHLA
                ************************************************************

MPXV_A.1        IVPTTRVDGNSYYWPDIYNSIIEAAINRGVKIRLLVGNWDKNDVYSMATARSLDALCVQN
MPXV_B.1        IVPTTRVDGNSYYWPDIYNSIIEAAINRGVKIRLLVGNWDKNDVYSMATARSLDALCVQN
VACV            IVPTTRVDGNSYYWPDIYNSIIEAAINRGVKIRLLVGNWDKNDVYSMATARSLDALCVQN
                ************************************************************

MPXV_A.1        DLSVKVFTIQNNTKLLIVDDEYVHITSANFDGTHYQNHGFVSFNSIDKQLVSEAKKIFER
MPXV_B.1        DLSVKVFTIQNNTKLLIVDDEYVHITSANFDGTHYQNHGFVSFNSIDKQLVSKAKKIFER
VACV            DLSVKVFTIQNNTKLLIVDDEYVHITSANFDGTHYQNHGFVSFNSIDKQLVSEAKKIFER
                ****************************************************:*******

MPXV_A.1        DWVSSHSKSLKI-
MPXV_B.1        DWVSSHSKSLKI-
VACV            DWVSSHSKSLKI-
                ************
```

Supplementary Data Fig 2. OPG057 (F13) sequence alignment

Amino acid sequence alignment of OPG057 (F13) from monkeypox virus (MPXV) Clade IIb lineages A.1 and B.1 and vaccinia virus (VACV). The E353K change found in lineage B.1 is highlighted in red.

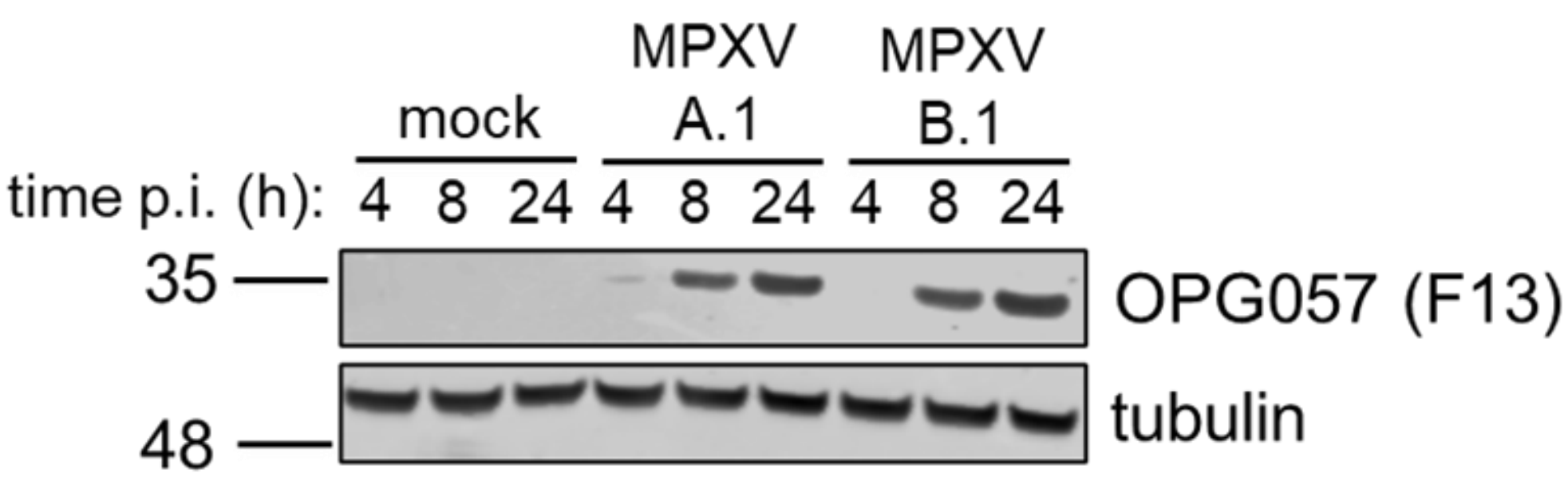

Supplementary Data Fig 3. OPG057 (F13) expression from MPXV Clade IIb lineages A.1 and B.1.

Immunoblot of human foetal foreskin fibroblasts (HFFF) cells mock-infected or infected at MOI 5 for 4, 8 or 24 h with the indicated viruses. Source data are provided at the end. MPXV: monkeypox virus. OPG: orthopoxvirus gene.

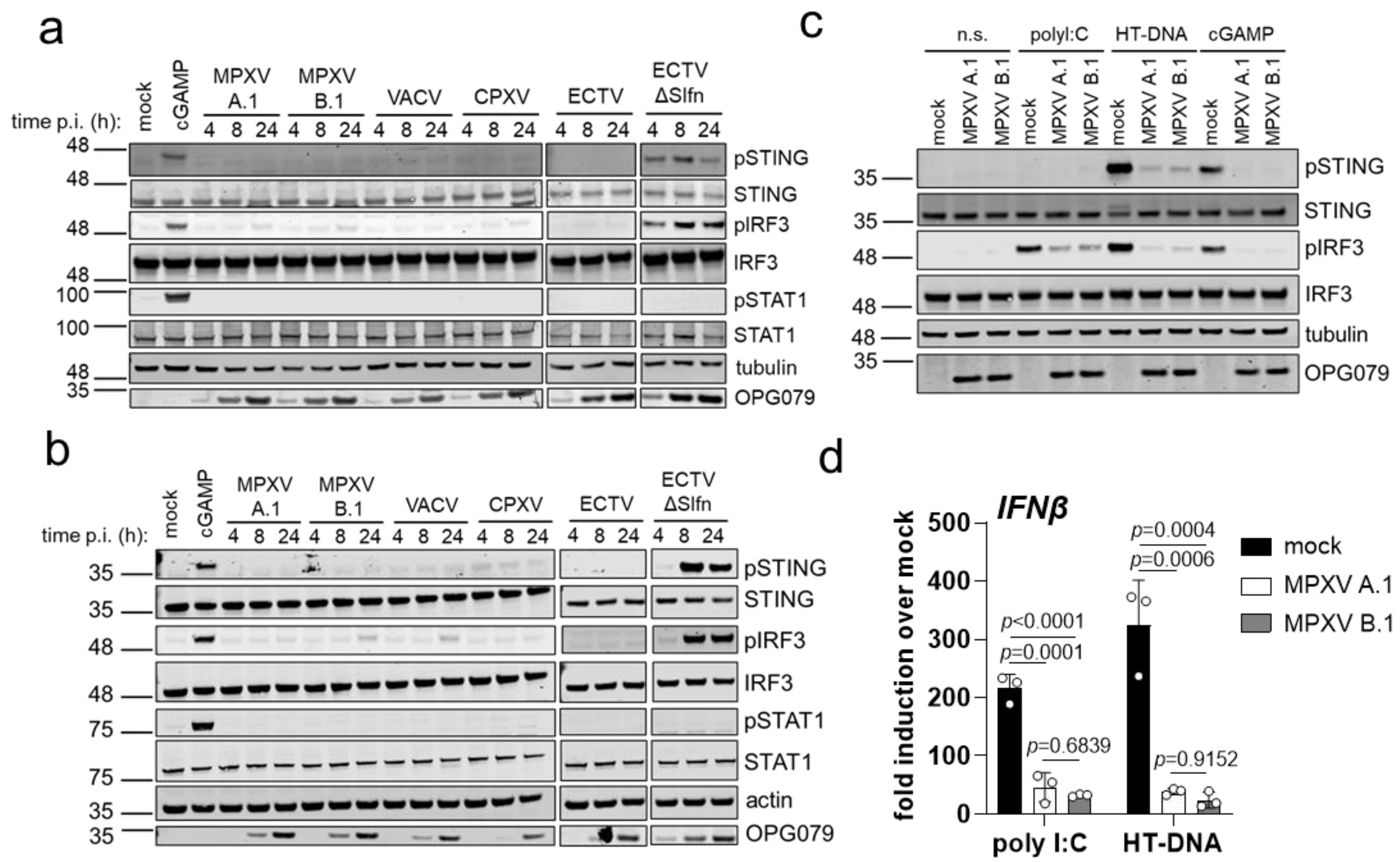

Supplementary Data Fig 4. MPXV lineages B.1 (global) and A.1 (endemic) evade innate sensing in human cells.

a, b Immunoblot of THP-1-derived macrophages (a) or human foetal foreskin fibroblasts (HFFF) (b) mock-infected or infected at MOI 5 for 4, 8 or 24 h with the indicated viruses, or transfected for 4 h with 1 µg/ml 2'3'-cGAMP as a control. **c**, Immunoblot of THP-1-derived macrophages infected for 6 h at MOI 5 with the indicated viruses and then either left non-stimulated (n.s.) or stimulated for 2 h by transfection with 1 µg/ml polyI:C, HT-DNA or cGAMP.

d, *IFNβ* expression measured by RT-qPCR from HFFF infected with MOI 5 of the indicated viruses for 4 h and then stimulated for 4 h by transfection with 500 ng/ml polyI:C or HT-DNA. Data are mean ± SD. n = 3 biological replicates per condition. Statistical analyses were performed using an ordinary one-way ANOVA with Tukey's multiple comparisons test. All data presented are representative of at least 2 experimental repeats. Source data are provided as a Source Data file. Source data for immunoblots are provided at the end. MPXV: monkeypox virus, VACV: vaccinia virus, CPXV: cowpox virus, ECTV: ectromelia virus.

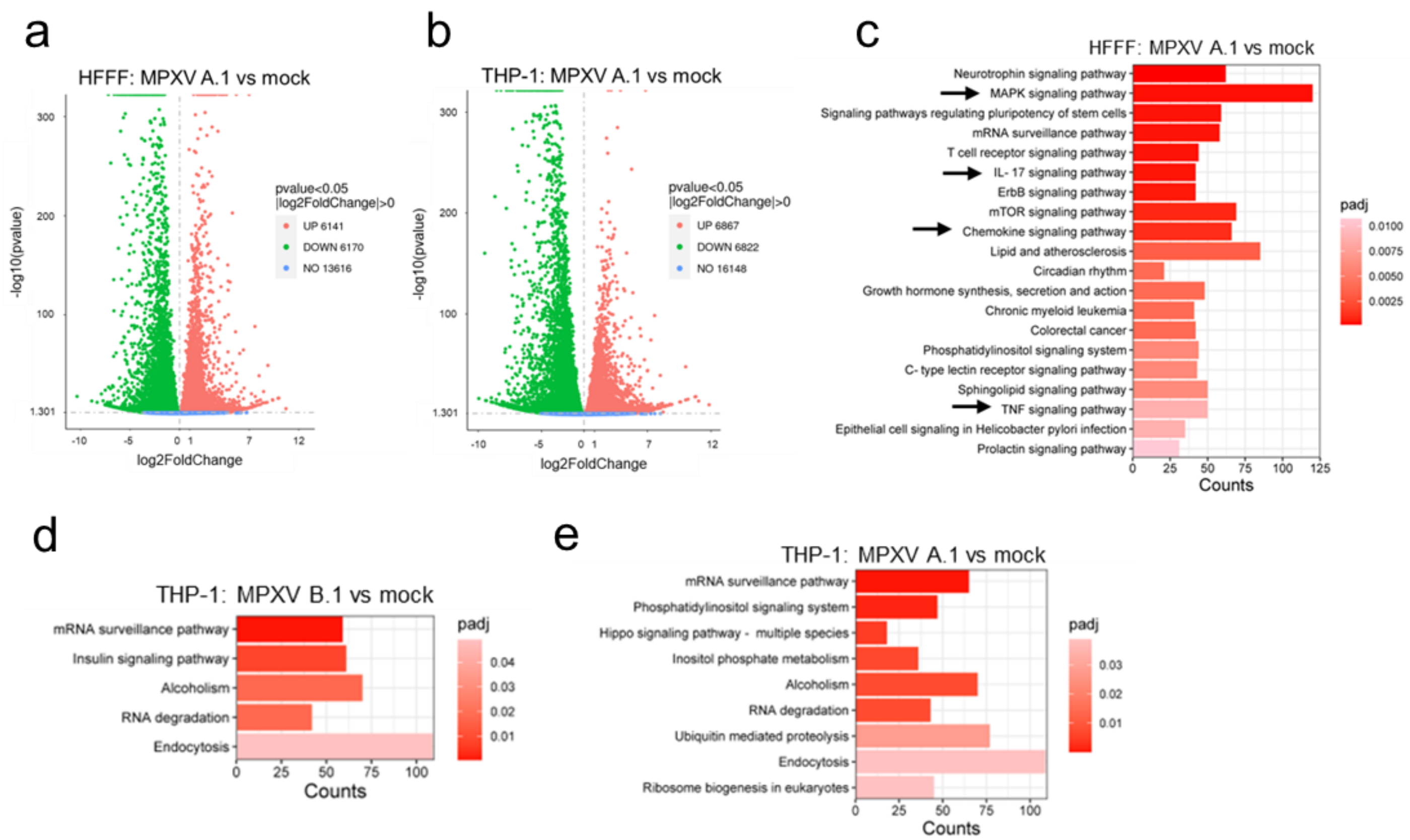

Supplementary Data Fig 5. Transcriptional changes during MPXV infection.

a, b, Significantly differentially expressed genes (log2FC>0.0) at 24 hpi by volcano plot analysis in human foetal foreskin fibroblasts (HFFF) (a) or THP-1-derived macrophages (b) infected with monkeypox virus (MPXV) A.1 at MOI 5, compared with the mock. *P* value determined by DESeq2.

c, Top 20 significantly upregulated pathways (KEGG analysis) from RNAseq data of HFFF cells infected with MOI 5 MPXV A.1 for 24 h compared with mock-infected cells. *P* value determined by DESeq2. Pathways of interest are highlighted with a black arrow.

d, e, Significantly upregulated pathways (KEGG analysis) in THP-1-derived macrophages infected with MOI 5 MPXV B.1 (**d**) or A.1 (**e**) compared with mock-infected cells. *P* value determined by DESeq2.

All data are from a single RNA-seq experiment.

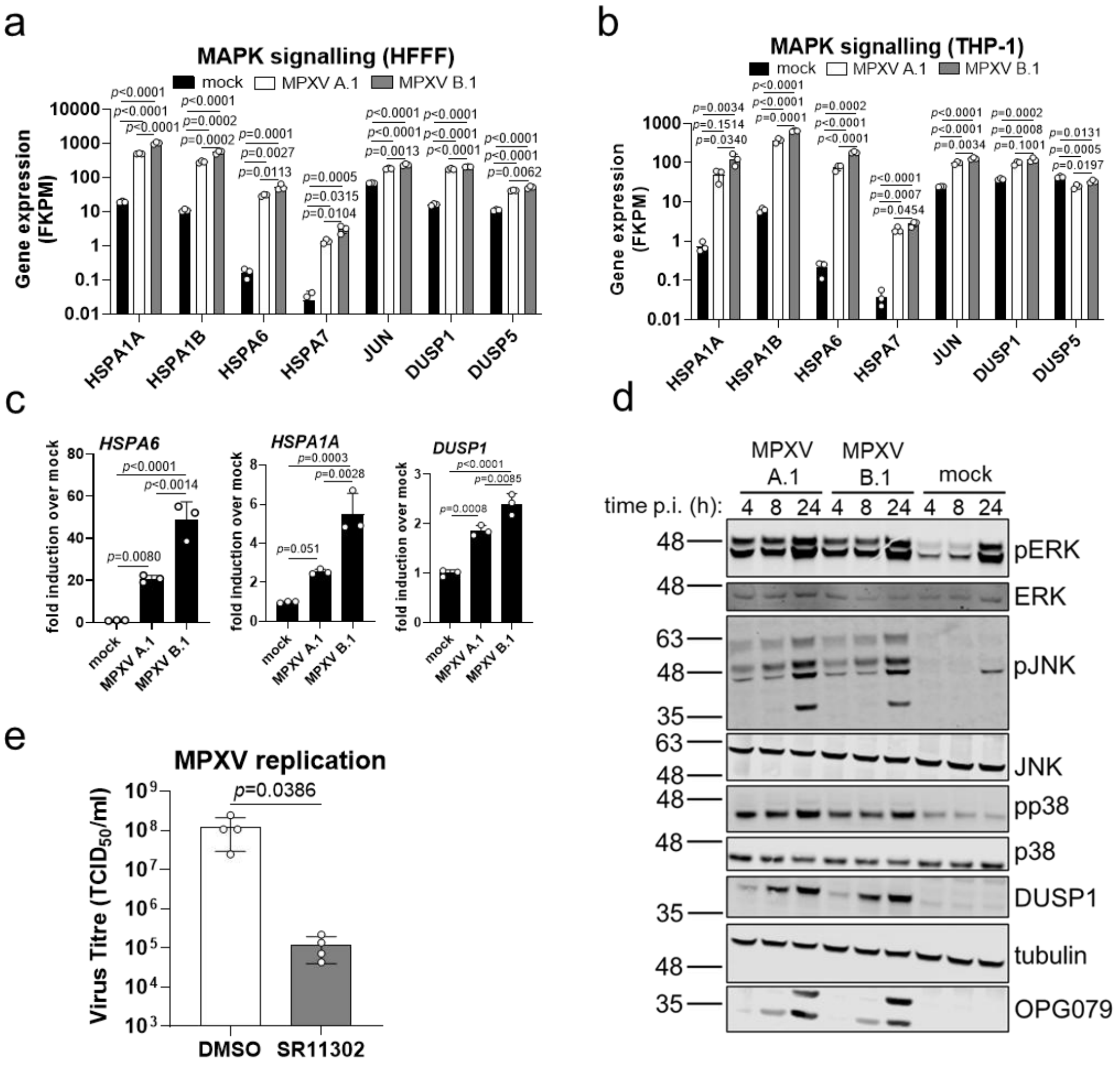

Supplementary Data Fig 6. MAPK pathway activation by MPXV lineages A.1 and B.1.

a, b, Expression level (FKPM) of a panel of mitogen-activated protein kinase (MAPK) signalling genes from infected human foetal foreskin fibroblasts (HFFF) (a) or THP-1-derived macrophages (b). Data are mean ± SD. n = 3 biological replicates per condition. Statistical analyses were performed using an ordinary one-way ANOVA with Tukey's multiple comparisons test.

c, RT-qPCR for MAPK signalling genes 24 hpi from HFFF infected with MOI 5 of the indicated viruses. Data are mean ± SD. n = 3 biological replicates per condition. Statistical analyses were performed using an ordinary one-way ANOVA with Tukey's multiple comparisons test.

d, Immunoblot of HFFF mock-infected or infected at MOI 5 for 4, 8 or 24 h with the indicated viruses.

e, MPXV B.1 virus titre at 48 h in HFFF in the presence or absence of 10 µM AP-1i inhibitor (SR11302). n = 4 biological replicates per condition. Data are mean ± SD. Statistical analyses were performed using an unpaired two-sided Student's t-test. All data presented are representative of at least 2 experimental repeats, except (a) and (b) that are from a single RNA-seq experiment. Source data are provided as a Source Data file. Source data for immunoblots are provided at the end. MPXV: monkeypox virus.

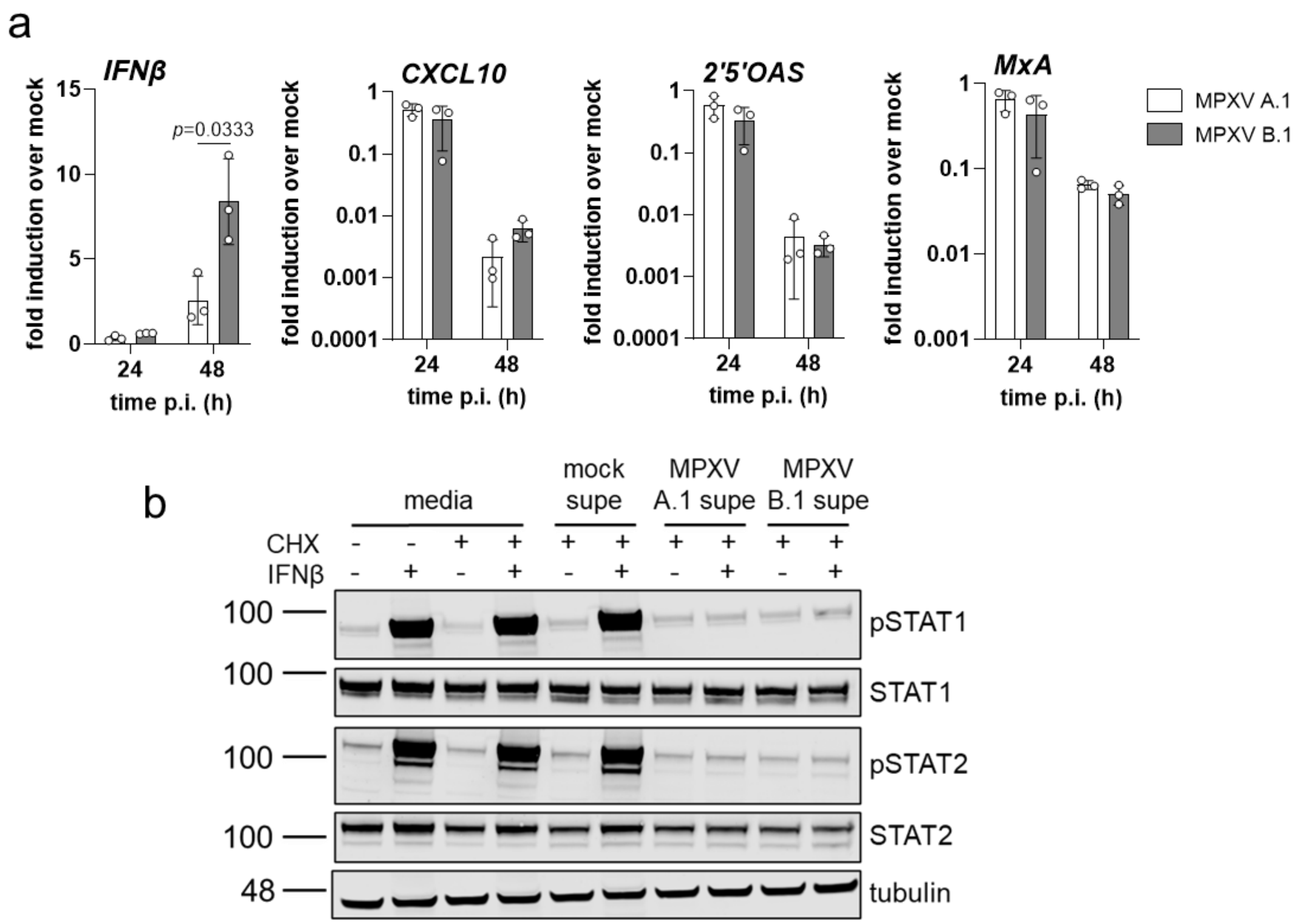

Supplementary Data Fig 7. MPXV lineage B.1, but not A.1, induces IFNβ expression in fibroblasts.

a, RT-qPCR for *IFNβ* and ISGs (*CXCL10*, *2'5'OAS* and *MxA*) at 24 and 48 hpi from human foetal foreskin fibroblasts (HFFF) infected with MOI 0.05 of the indicated viruses. Data are mean ± SD. n = 3 biological replicates per condition. Statistical analyses were performed using an unpaired two-sided Student's t-test.

b, Immunoblot of THP-1-derived macrophages stimulated for 2 h with 0.5 ng/ml IFNβ -/+ cycloheximide (CHX, 25 μM) in the presence of supernatant (supe) from mock or monkeypox virus (MPXV)-infected cells (HFFF, 24 h, MOI 5) or fresh media as a control.

Data presented are representative of 2 experimental repeats. Source data are provided as a Source Data file. Source data for immunoblots are provided at the end.

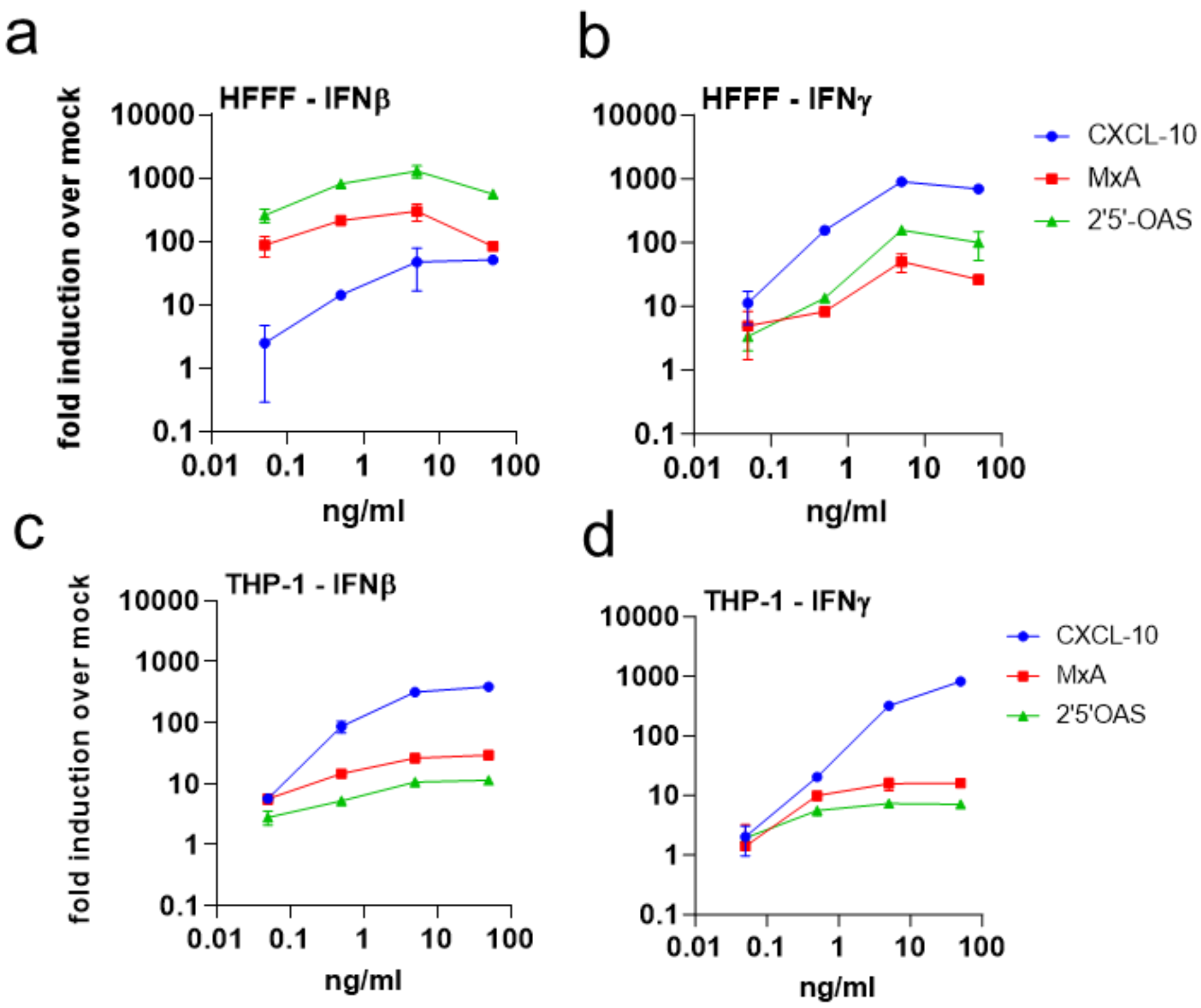

Supplementary Data Fig 8. ISG responses in HFFF and THP-1-derived macrophages treated with type I or type II IFN.

a-d, RT-qPCR for ISGs *CXCL-10*, *MxA* and *2'5'OAS* from human foetal foreskin fibroblasts (HFFF) (**a, b**) or THP-1-derived macrophages (**c, d**) treated for 16 h with the indicated doses of type I (IFNβ) (a, c) or type II (IFNγ) (b, d) IFN. All data are mean ± SD, n = 3 biological repeats per condition.

Data presented are representative of 2 experimental repeats. Source data are provided as a Source Data file.

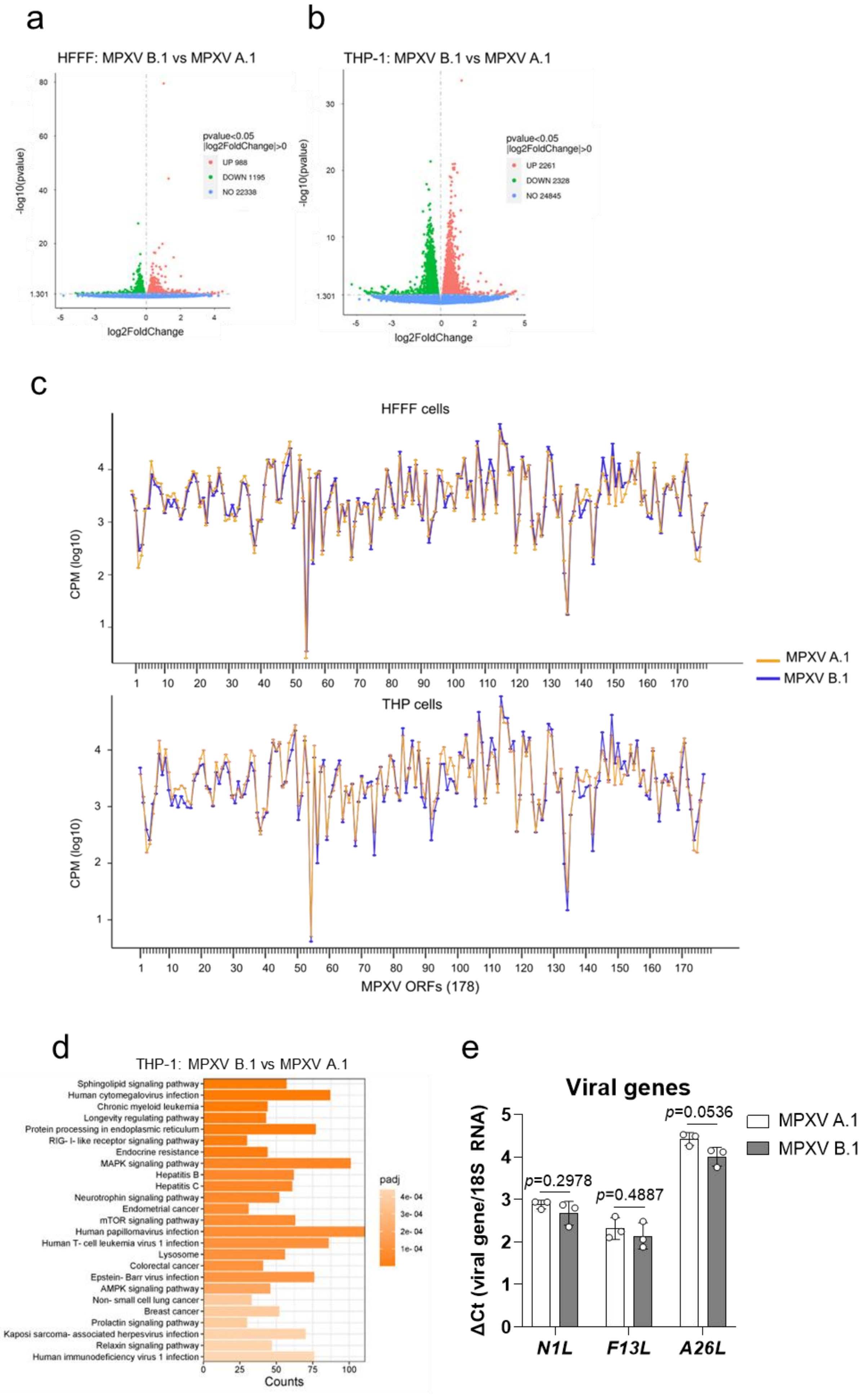
a
HFFF: MPXV B.1 vs MPXV A.1
-log10(pvalue)
log2FoldChange
pvalue<0.05
|log2FoldChange|>0
UP 988
DOWN 1195
NO 22338
b
THP-1: MPXV B.1 vs MPXV A.1
-log10(pvalue)
log2FoldChange
pvalue<0.05
|log2FoldChange|>0
UP 2261
DOWN 2328
NO 24845
c
HFFF cells
THP cells
CPM (log10)
MPXV ORFs (178)
MPXV A.1
MPXV B.1
d
THP-1: MPXV B.1 vs MPXV A.1
Sphingolipid signaling pathway
Human cytomegalovirus infection
Chronic myeloid leukemia
Longevity regulating pathway
Protein processing in endoplasmic reticulum
RIG- I- like receptor signaling pathway
Endocrine resistance
MAPK signaling pathway
Hepatitis B
Hepatitis C
Neurotrophin signaling pathway
Endometrial cancer
mTOR signaling pathway
Human papillomavirus infection
Human T- cell leukemia virus 1 infection
Lysosome
Colorectal cancer
Epstein- Barr virus infection
AMPK signaling pathway
Non- small cell lung cancer
Breast cancer
Prolactin signaling pathway
Kaposi sarcoma- associated herpesvirus infection
Relaxin signaling pathway
Human immunodeficiency virus 1 infection
Counts
padj
e
Viral genes
ΔCt (viral gene/18S RNA)
p=0.2978
p=0.4887
p=0.0536
N1L
F13L
A26L
MPXV A.1
MPXV B.1

Supplementary Data Fig 9. Differential innate immune control by MPXV lineages B.1 and A.1.

a, b, Significantly differentially expressed genes (log2FC>0.0) in human foetal foreskin fibroblasts (HFFF) (**a**) and THP-1-derived macrophages (**b**) by volcano plot analysis at 24 hpi with monkeypox virus (MPXV) B.1 at MOI 5 compared with MPXV A.1 infection. *P* value determined by DESeq2.

c, Counts per million (CPM) per MPXV open reading frame (ORF) from RNA seq data.

d, Top 25 differentially regulated pathways (KEGG analysis) from RNAseq data of THP-1-derived macrophages infected with MOI 5 MPXV B.1 for 24 h compared with MPXV A.1-infected cells. *P* value determined by DESeq2.

e, Viral gene expression by RT-qPCR at 8 h post-infection from cells in Fig 4c measuring representative early (*OPG035)*, intermediate (*OPG057)* and late (*OPG153)* viral transcripts. Data are mean ± SD. n = 3 biological repeats per condition. Statistical analyses were performed using an unpaired two-sided Student's t-test.

Data presented are from a single RNA-seq experiment, except (e) that is a representative experiment of 2 repeats. Source data are provided as a Source Data file.

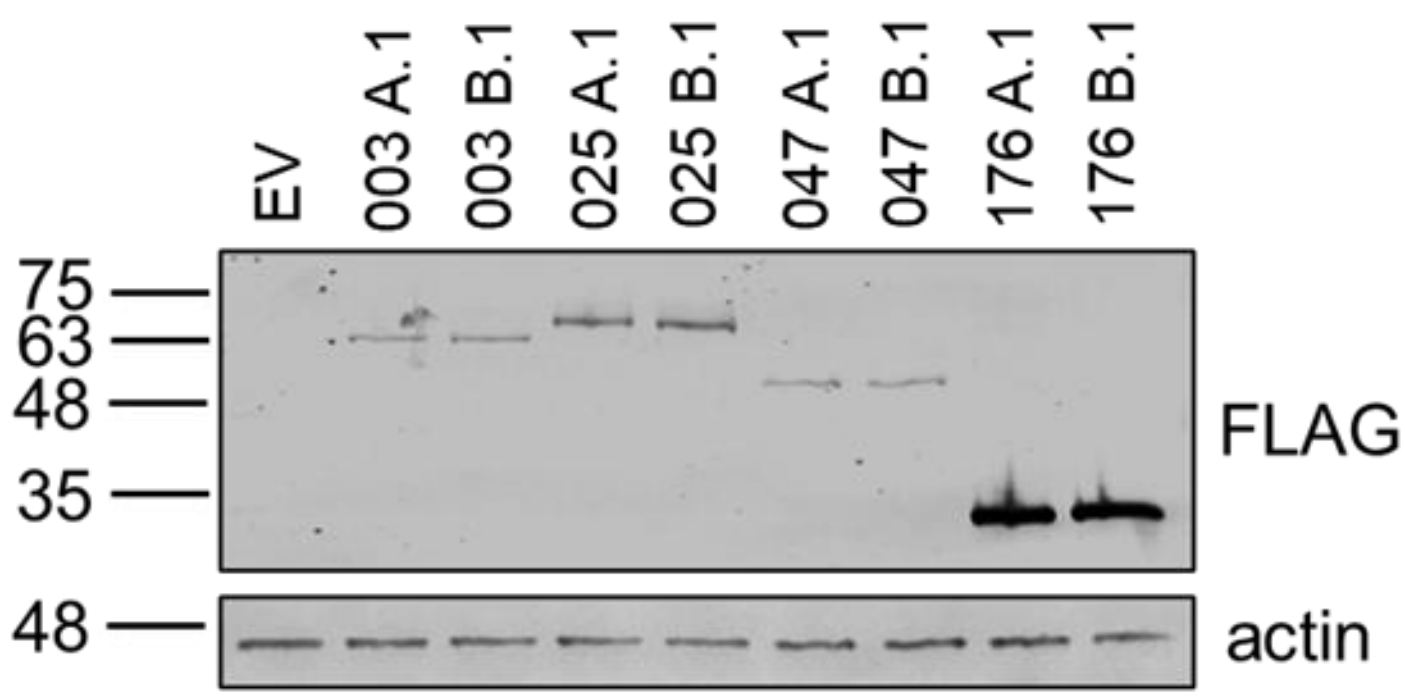

Supplementary Data Fig 10: MPXV B.1 OPG047 has a reduced capacity to suppress NF-κB activation.

Immunoblotting of FLAG-tagged orthopoxvirus gene (OPG) allele expression from the reporter gene assay in Fig 4d. Source data are provided at the end.

Supplementary Table 1

Single nucleotide differences between MPXV B.1 strain CVR_S1 (accession number ON808413) and MPXV A.1 strain UK_P3 (accession number MT903345)

Position	A.1 nt	B.1 nt	A. 1 aa	B.1 aa	OPG gene	VACV COP gene	Description
1284	G	A	S	L	OPG001	C23L	secreted chemokine binding protein
2613	G	A	S	F	OPG002	C22L	secreted TNFα receptor like protein
3133	G	A	silent		OPG003	C19L	ankyrin repeat protein
3544	G	A	silent		OPG003	C19L	ankyrin repeat protein
3840	C	T	D	N	OPG003	C19L	ankyrin repeat protein
7793	C	T	silent		OPG019	C11R	EGF like domain protein
14022	G	T	A	D	OPG025	C9L	ankyrin repeat protein
15450	G	A	intergenic				
21745	G	A	silent		OPG037	M1L	ankyrin-like protein
30389	G	A	silent		OPG047	F3L	kelch-like protein
31075	G	A	R	C	OPG047	F3L	kelch-like protein
34481	G	A	P	S	OPG053	F9L	IMV membrane protein
37224	G	A	silent		OPG056	F12L	EEV maturation protein
38382	G	A	silent		OPG056	F12L	EEV maturation protein
38684	C	T	E	K	OPG056	F12L	EEV maturation protein
39161	C	T	E	K	OPG057	F13L	Palmytilated EEV membrane protein
52907	G	A	silent		OPG071	E9L	DNA polymerase
54139	G	A	L	F	OPG071	E9L	DNA polymerase
54657	G	A	D	N	OPG072	E10R	sulfhydryl oxidase
64319	G	A	silent		OPG083	I7L	virion core cysteine protease
73088	C	T	S	L	OPG093	G8R	VLTF-1 late transcription factor
73261	G	A	D	N	OPG093	G8R	VLTF-1 late transcription factor
74227	G	A	M	I	OPG094	G9R	entry/fusion complex component
77405	G	A	E	K	OPG098	L4R	ss/dsDNA binding protein
81297	G	A	silent		OPG105	J6R	RNA polymerase subunit (RPO147)
82395	C	T	silent		OPG105	J6R	RNA polymerase subunit (RPO147)
82473	G	A	silent		OPG105	J6R	RNA polymerase subunit (RPO147)
84609	C	T	silent		OPG105	J6R	RNA polymerase subunit (RPO147)
95056	G	A	silent		OPG115	D3R	virion core protein
124152	G	A	E	K	OPG145	A18R	DNA helicase
124696	G	A	R	Q	OPG145	A18R	DNA helicase
128720	C	T	S	L	OPG150	A23R	intermediate transcription factor VITF-3
150494	C	T	H	Y	OPG176	A46R	IL-1/TLR signaling inhibitor
151486	A	C	intergenic				
155820	G	A	intergenic				
162356	C	T	silent		OPG188	B2R	viral schlafen
170287	G	A	silent		OPG198	B12R	Ser/Thr Kinase
178204	G	A	intergenic				
181997	G	A	D	N	OPG210	B21R	surface glycoprotein
183536	C	T	P	S	OPG210	B21R	surface glycoprotein
186696	G	A	M	I	OPG210	B21R	surface glycoprotein
193409	G	A	D	N	OPG003	C19L	ankyrin repeat protein
193705	C	T	silent		OPG003	C19L	ankyrin repeat protein
194116	C	T	silent		OPG003	C19L	ankyrin repeat protein

194636	C	T	S	F	OPG002	C22L	secreted TNFα receptor like protein
195965	C	T	S	L	OPG001	C23L	secreted chemokine binding protein

Suppl Fig 1e

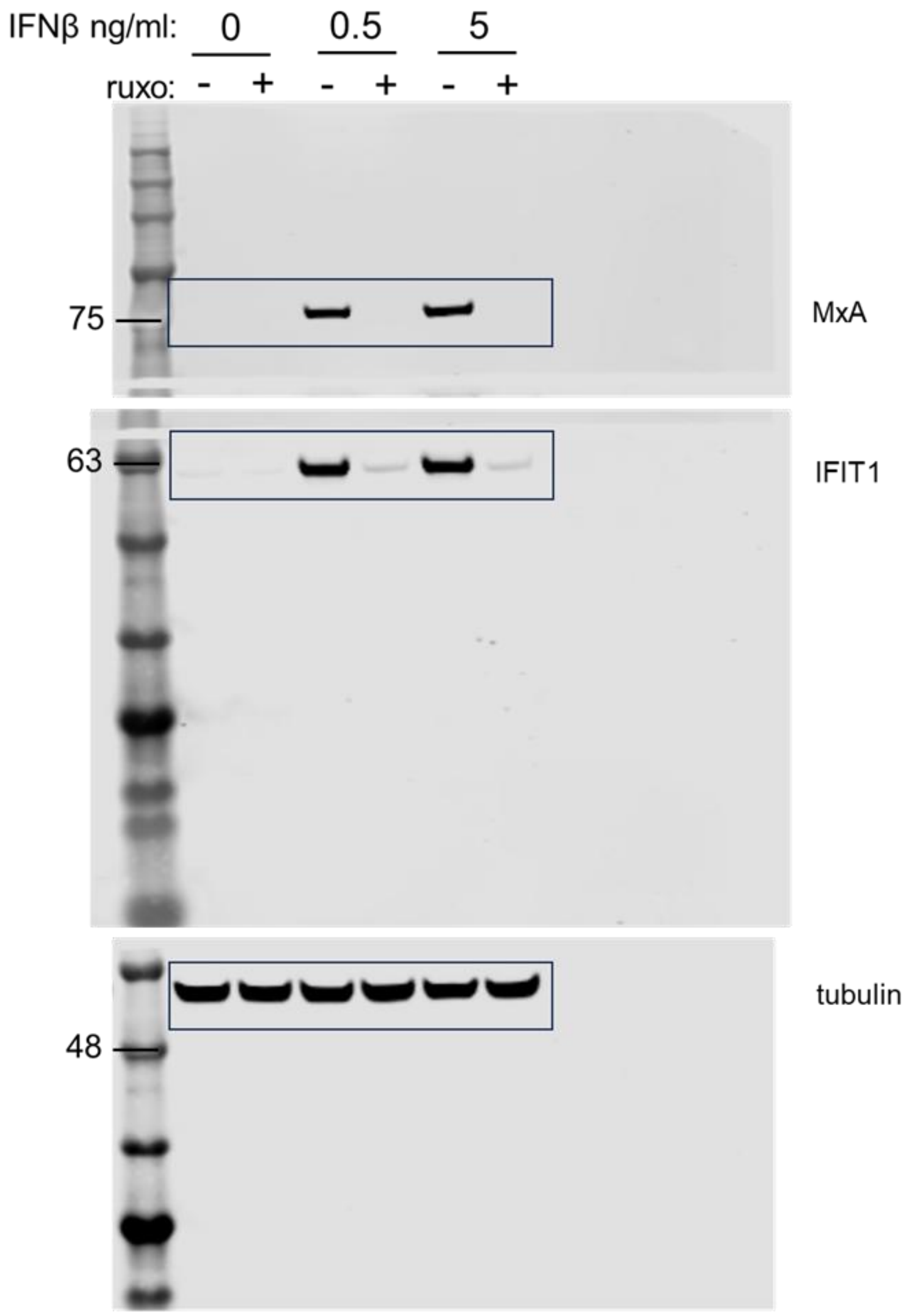

Suppl Fig 1g

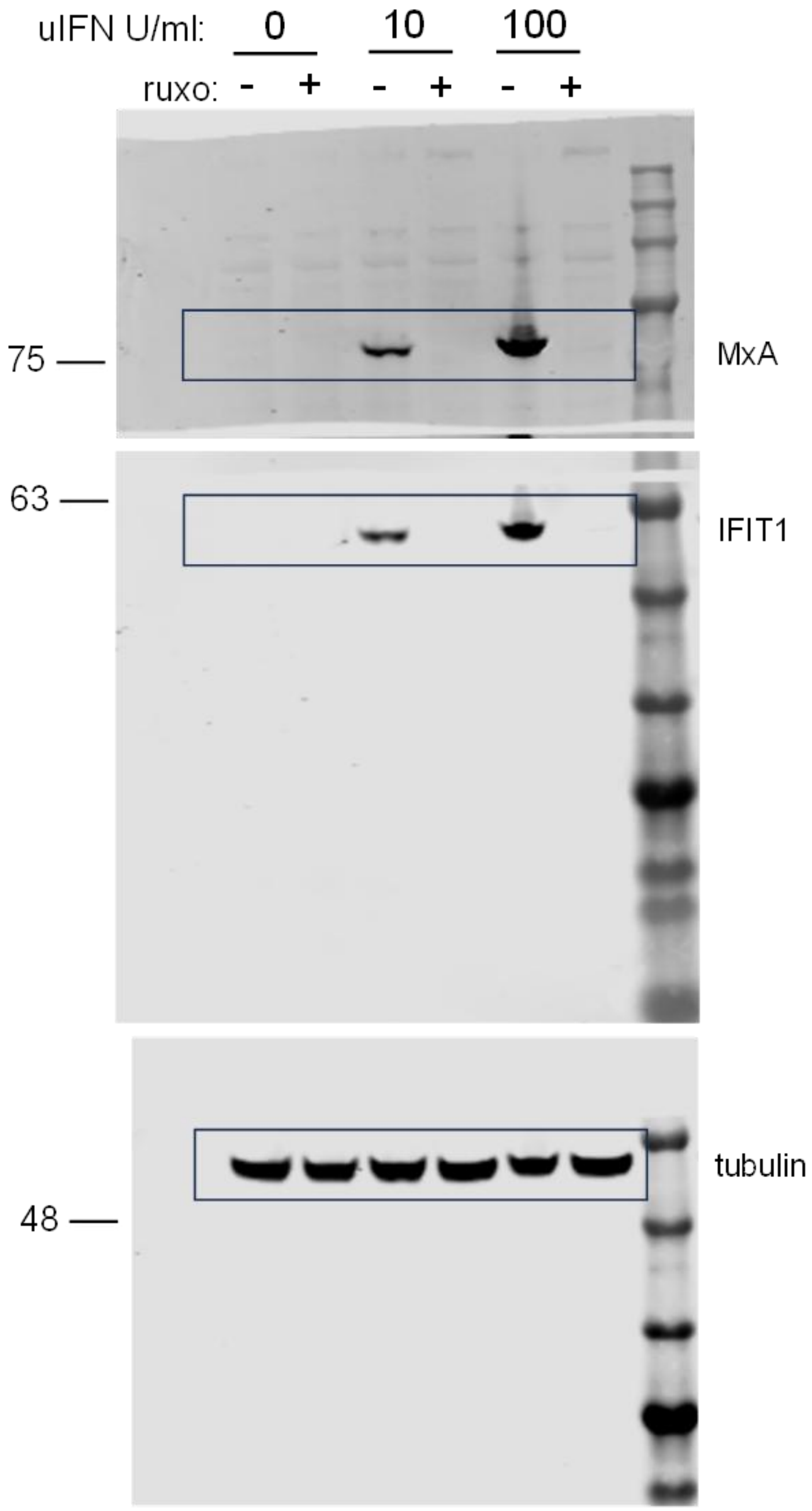

Suppl Fig 3

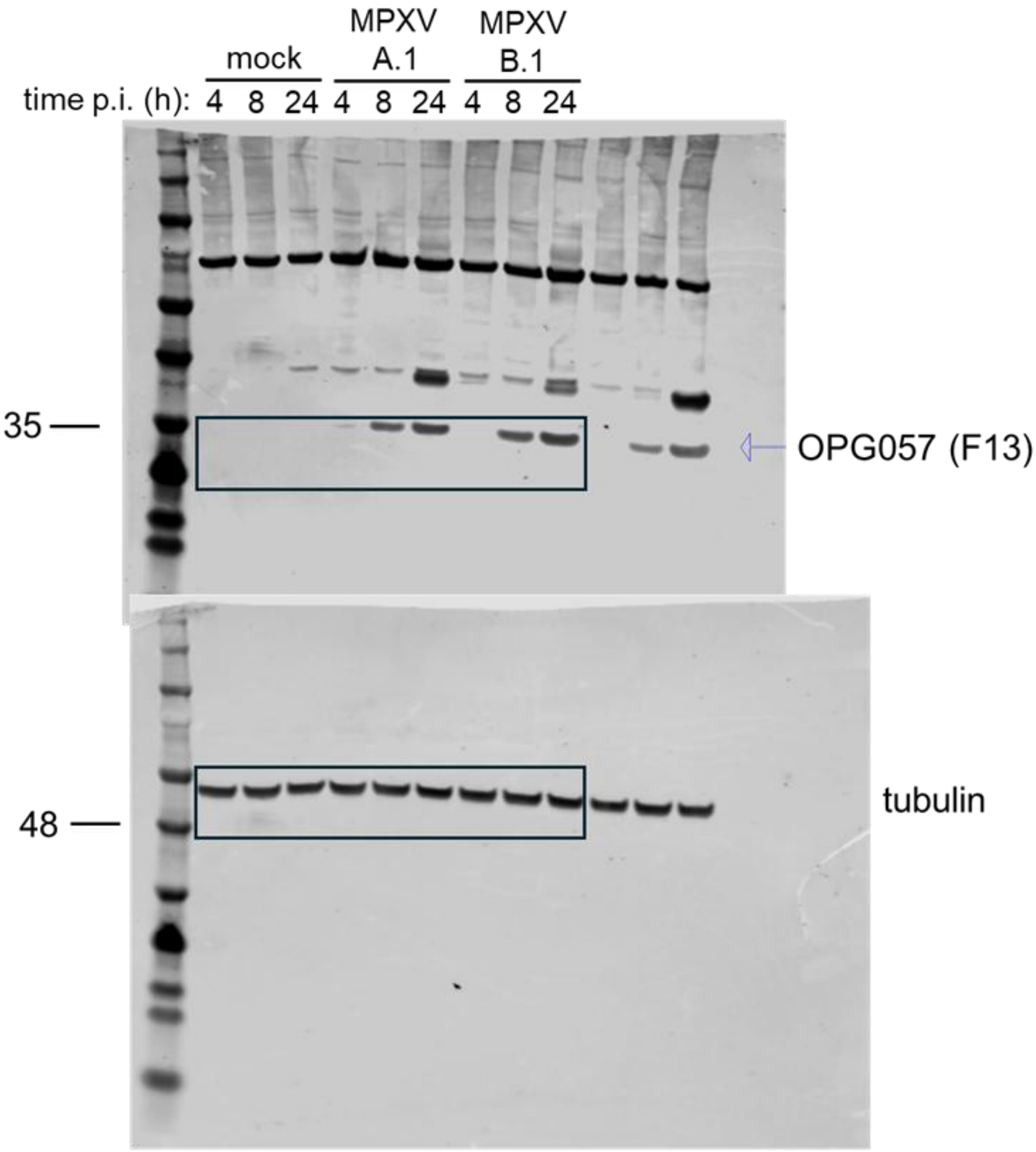

mock
MPXV A.1
MPXV B.1
time p.i. (h): 4 8 24 4 8 24 4 8 24
35
OPG057 (F13)
48
tubulin

Suppl Fig 4a

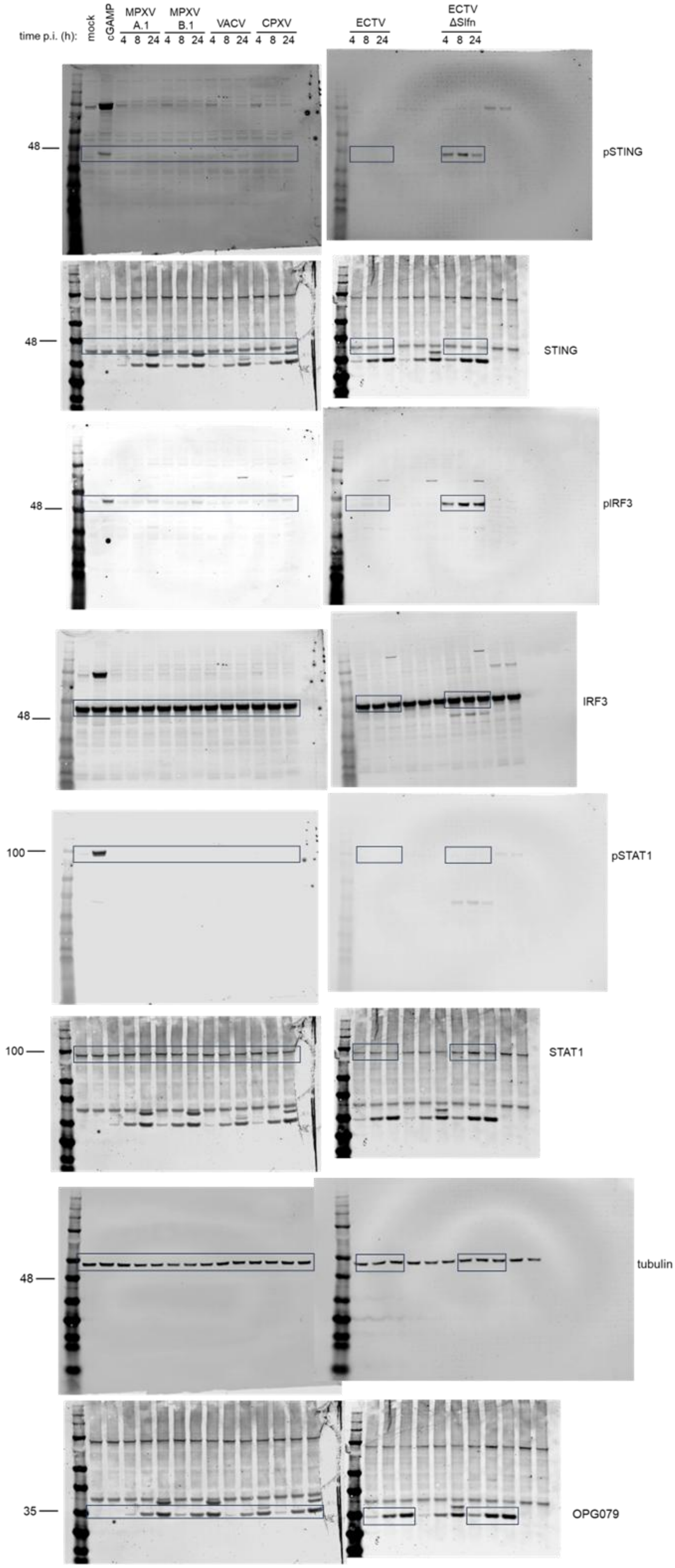

time p.i. (h):
mock
cGAMP
MPXV A.1
MPXV B.1
VACV
CPXV
4 8 24
ECTV
ECTV ΔSlfn
48
pSTING
STING
pIRF3
IRF3
100
pSTAT1
STAT1
tubulin
35
OPG079

Suppl Fig 4b

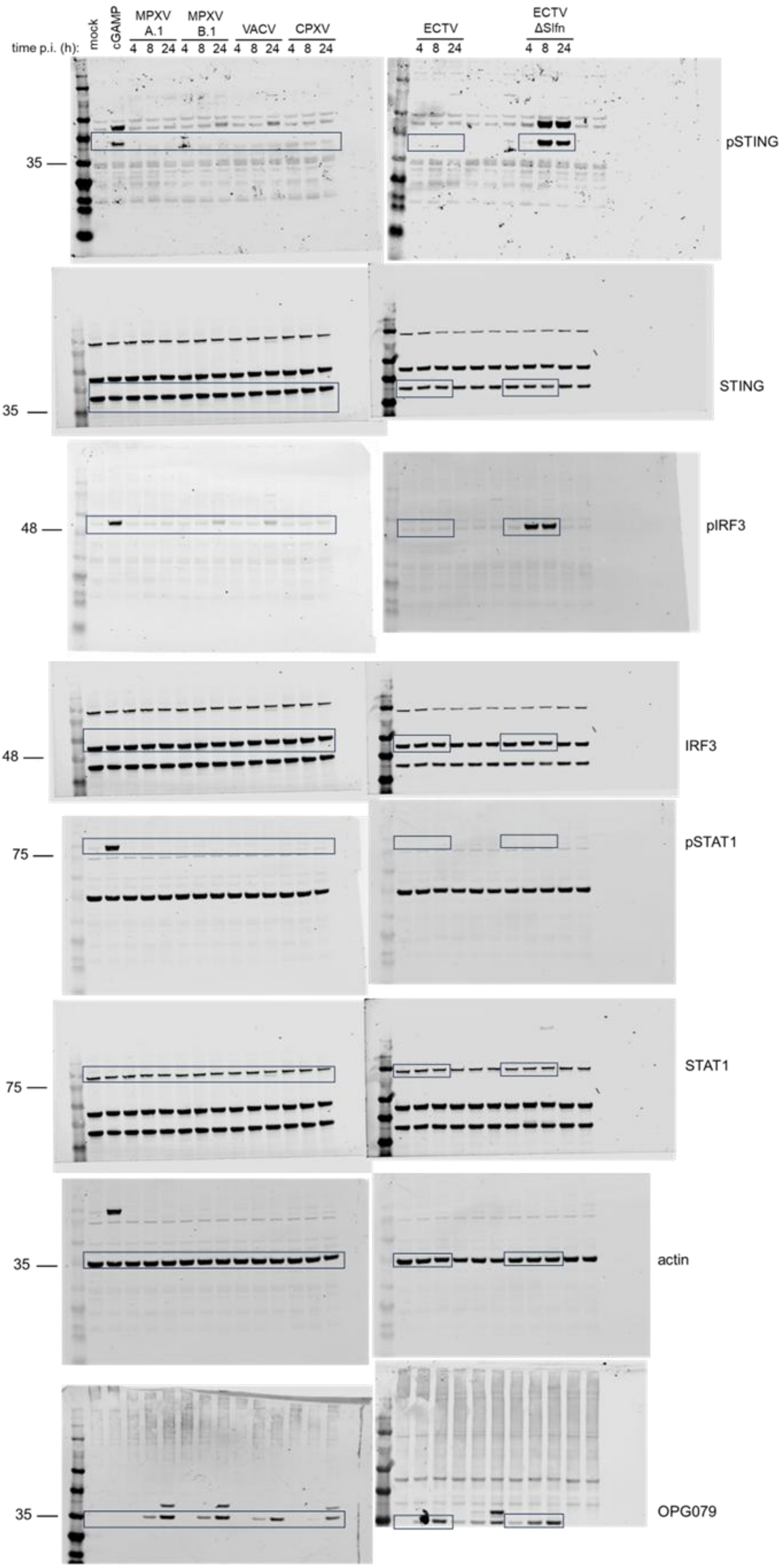
time p.i. (h):
mock
cGAMP
MPXV A.1
MPXV B.1
VACV
CPXV
4 8 24 4 8 24 4 8 24 4 8 24
ECTV
ECTV ΔSlfn
4 8 24
4 8 24
35
pSTING
35
STING
48
pIRF3
48
IRF3
75
pSTAT1
75
STAT1
35
actin
35
OPG079

Suppl Fig 4c

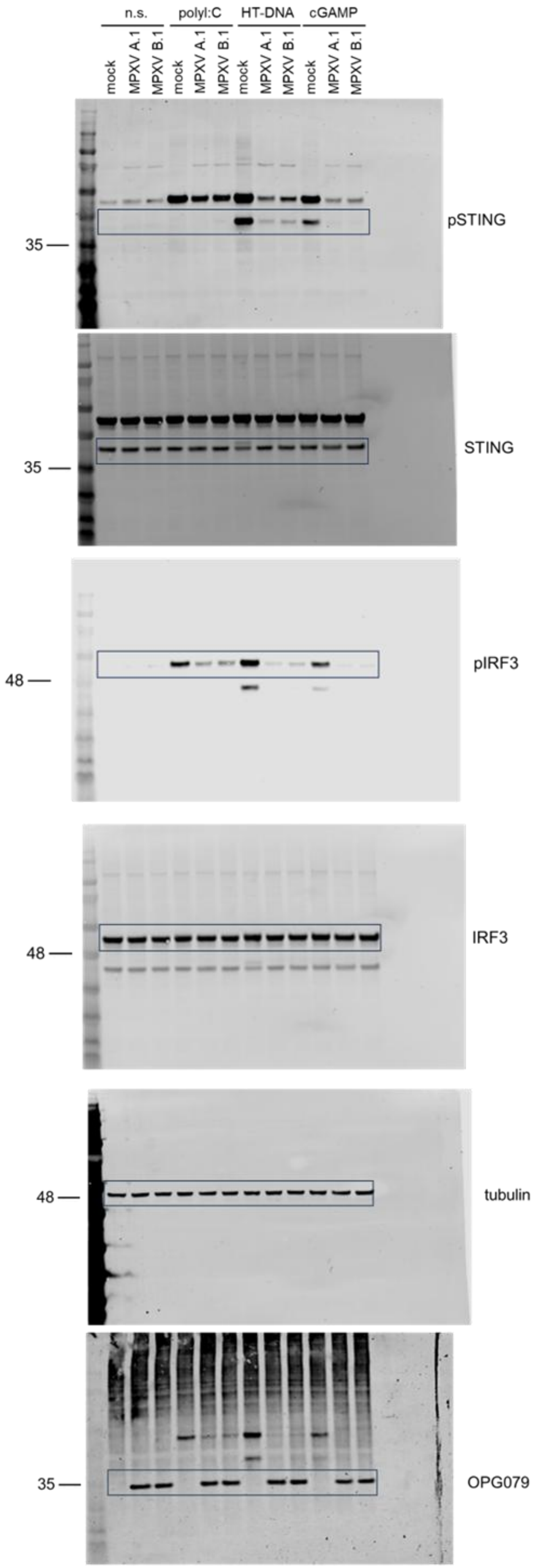
n.s.
polyI:C
HT-DNA
cGAMP
mock
MPXV A.1
MPXV B.1
mock
MPXV A.1
MPXV B.1
mock
MPXV A.1
MPXV B.1
mock
MPXV A.1
MPXV B.1
35
pSTING
35
STING
48
pIRF3
48
IRF3
48
tubulin
35
OPG079

Suppl Fig 6d

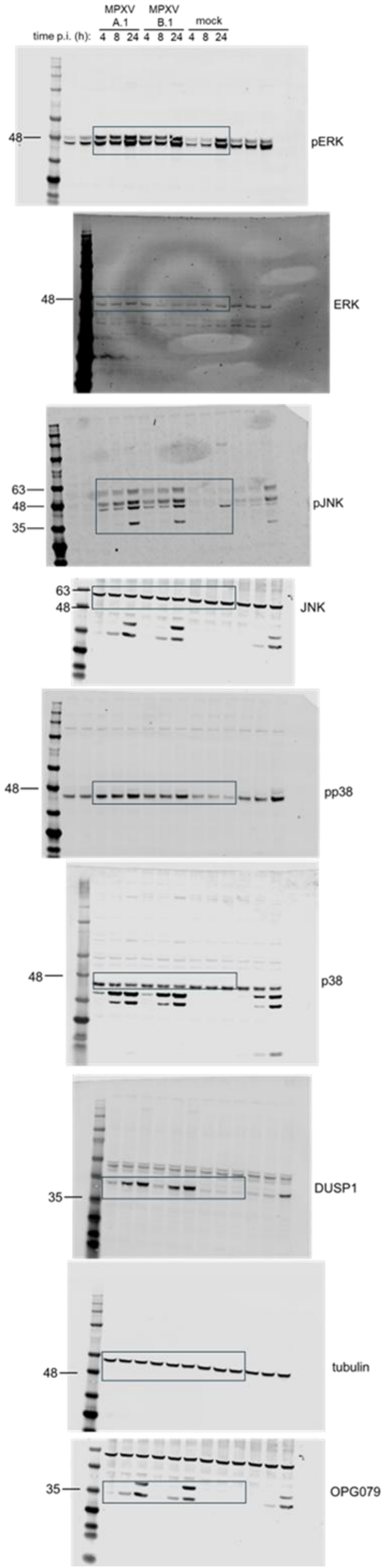
MPXV A.1
MPXV B.1
mock
time p.i. (h): 4 8 24 4 8 24 4 8 24
48
pERK
48
ERK
63
48
35
pJNK
63
48
JNK
48
pp38
48
p38
35
DUSP1
48
tubulin
35
OPG079

Suppl Fig 7b

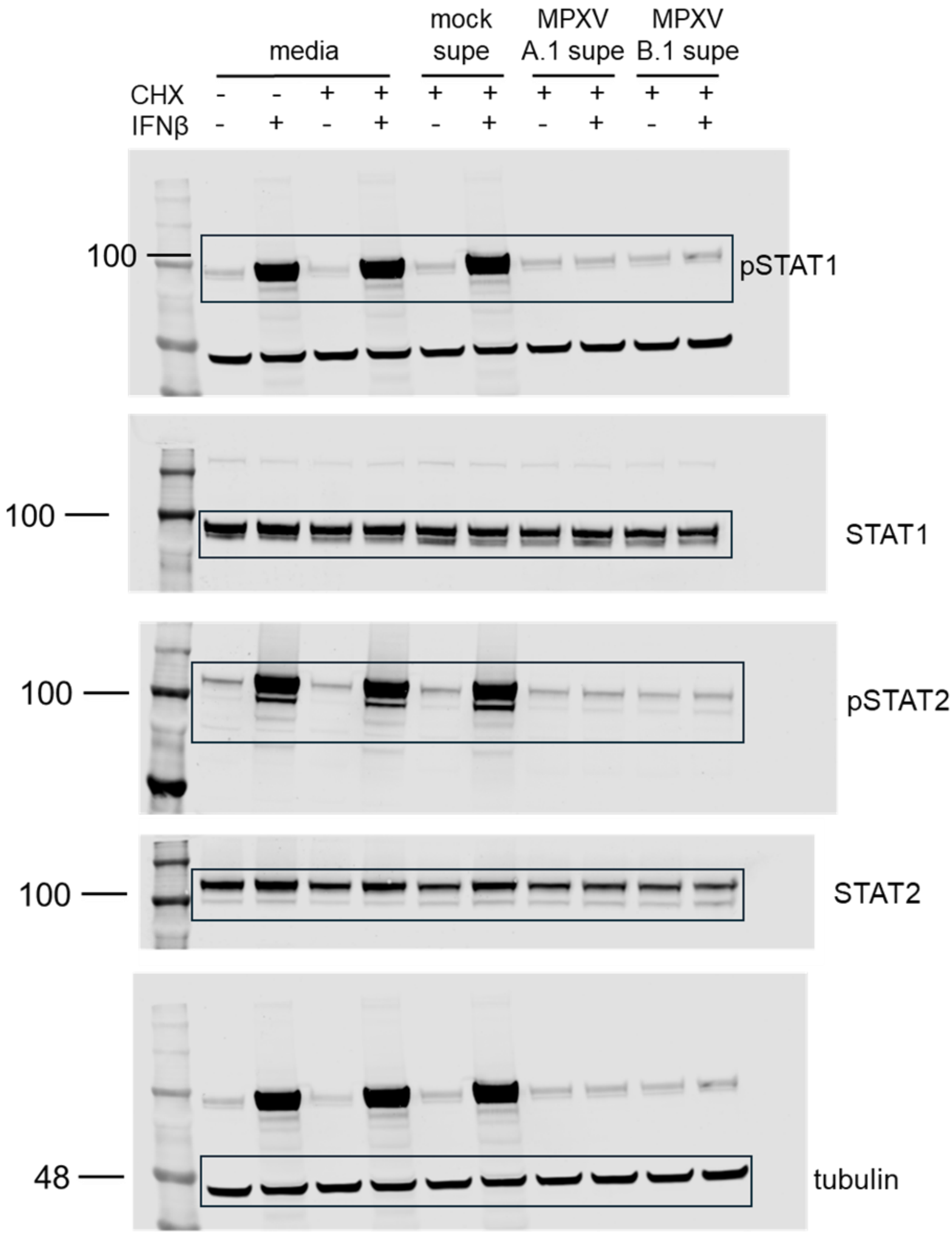

media
mock supe
MPXV A.1 supe
MPXV B.1 supe
CHX - - + + + + + + + +
IFNβ - + - + - + - + - +
100
pSTAT1
100
STAT1
100
pSTAT2
100
STAT2
48
tubulin

Suppl Fig 10

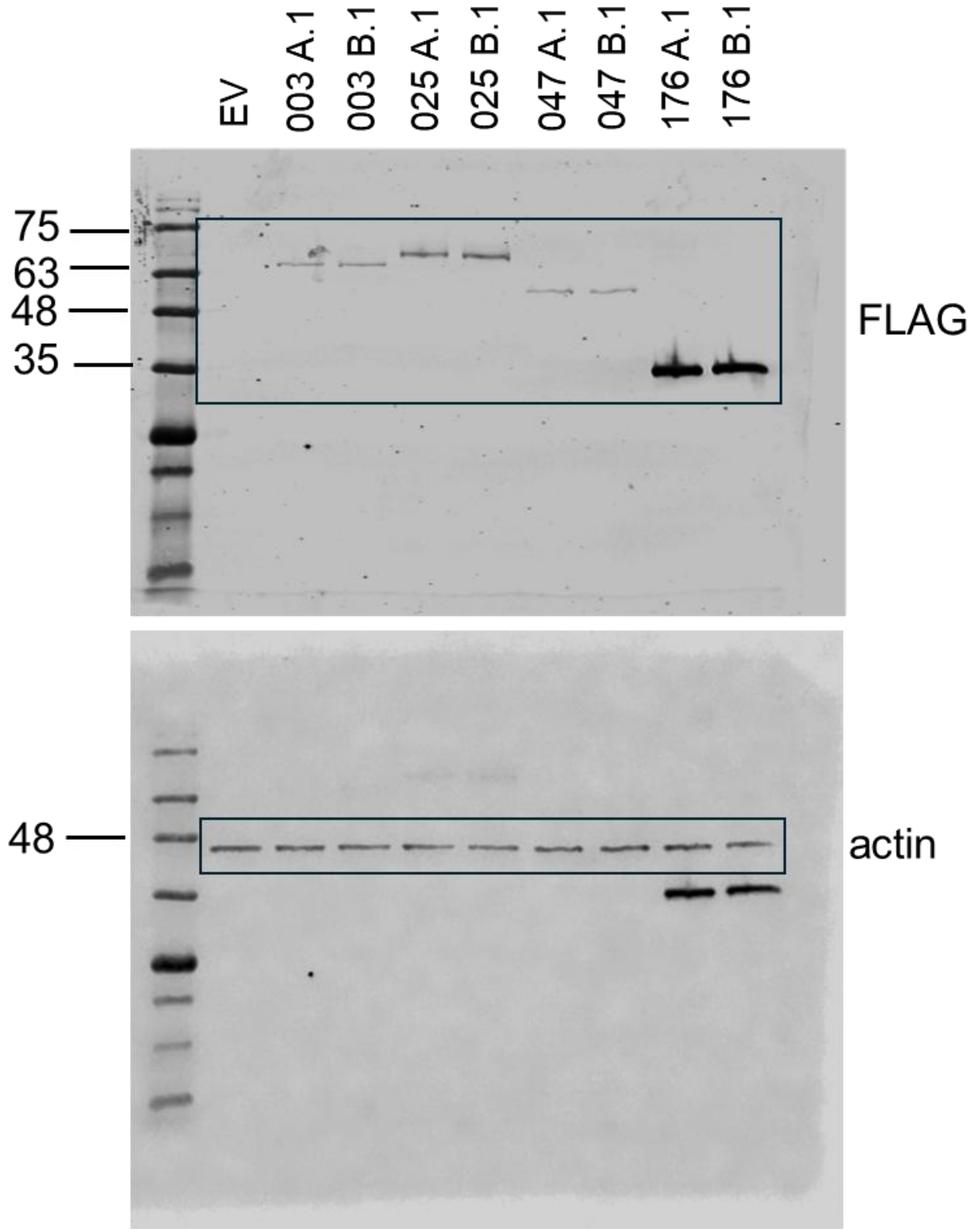
EV
003 A.1
003 B.1
025 A.1
025 B.1
047 A.1
047 B.1
176 A.1
176 B.1
75
63
48
35
FLAG
48
actin
