# Supplementary material for: APOBEC3-driven attenuation of the 2022 global outbreak monkeypox virus relative to its clade IIb ancestor
Source: Nat Commun. 2026 Aug 11;17:9630. doi: 10.1038/s41467-026-76580-7 (PMC13554265; doi:10.1038/s41467-026-76580-7)
Supplement: Supplementary file 2 [file 41467_2026_76580_MOESM2_ESM.pdf]

nature portfolio

Corresponding author(s): Carlos Maluquer de Motes

Last updated by author(s): Jul 6, 2026

Reporting Summary

Nature Portfolio wishes to improve the reproducibility of the work that we publish. This form provides structure for consistency and transparency in reporting. For further information on Nature Portfolio policies, see our Editorial Policies and the Editorial Policy Checklist.

Statistics

For all statistical analyses, confirm that the following items are present in the figure legend, table legend, main text, or Methods section.

n/a	Confirmed	
☐	☒	The exact sample size (*n*) for each experimental group/condition, given as a discrete number and unit of measurement
☐	☒	A statement on whether measurements were taken from distinct samples or whether the same sample was measured repeatedly
☐	☒	The statistical test(s) used AND whether they are one- or two-sided<br>*Only common tests should be described solely by name; describe more complex techniques in the Methods section.*
☐	☒	A description of all covariates tested
☐	☒	A description of any assumptions or corrections, such as tests of normality and adjustment for multiple comparisons
☐	☒	A full description of the statistical parameters including central tendency (e.g. means) or other basic estimates (e.g. regression coefficient) AND variation (e.g. standard deviation) or associated estimates of uncertainty (e.g. confidence intervals)
☐	☒	For null hypothesis testing, the test statistic (e.g. *F*, *t*, *r*) with confidence intervals, effect sizes, degrees of freedom and *P* value noted<br>*Give P values as exact values whenever suitable.*
☒	☐	For Bayesian analysis, information on the choice of priors and Markov chain Monte Carlo settings
☒	☐	For hierarchical and complex designs, identification of the appropriate level for tests and full reporting of outcomes
☒	☐	Estimates of effect sizes (e.g. Cohen's *d*, Pearson's *r*), indicating how they were calculated

Our web collection on statistics for biologists contains articles on many of the points above.

Software and code

Policy information about availability of computer code

Data collection	ImageStudio Acquisition Software (version 5.2)<br>CLARIOstar software (version 5.20 RS)<br>Quantstudio design and analysis software (version 1.5.2)
Data analysis	Commercial programs used in this study include GraphPad Prism (version 10.1.2), Image Studio Lite Quantification software (version 5.5.4) and MARS Data analysis software (version 2.00).<br>Open-source programs used in this study include HISAT2 (version 2.1.0), featureCounts (version 1.5.0), DESeq2 (version 3.20), ImageJ-Fiji (version 1.53)

For manuscripts utilizing custom algorithms or software that are central to the research but not yet described in published literature, software must be made available to editors and reviewers. We strongly encourage code deposition in a community repository (e.g. GitHub). See the Nature Portfolio guidelines for submitting code & software for further information.

Data

Policy information about availability of data

All manuscripts must include a data availability statement. This statement should provide the following information, where applicable:

- Accession codes, unique identifiers, or web links for publicly available datasets
- A description of any restrictions on data availability
- For clinical datasets or third party data, please ensure that the statement adheres to our policy

All data included in this study including supplementary material will be freely available. RNA-seq data have been uploaded in the Gene Expression Omnibus repository, with the dataset identifier GSE338258. OPXV nucleotide sequences cited in this study are available on NCBI GenBank: MPXV_CVR_S1 (ON808413), MPXV_CBM (GCA_964276875), MPXV_UK_P2 (MT903344), MPXV_UK_P3 (MT903345), VACV Western Reserve (AY243312), CPXV Brighton-Red (AF482758.2) and ECTV Moscow (AF012825).

Research involving human participants, their data, or biological material

Policy information about studies with human participants or human data. See also policy information about sex, gender (identity/presentation), and sexual orientation and race, ethnicity and racism.

Reporting on sex and gender	N/A
Reporting on race, ethnicity, or other socially relevant groupings	N/A
Population characteristics	N/A
Recruitment	N/A
Ethics oversight	N/A

Note that full information on the approval of the study protocol must also be provided in the manuscript.

Field-specific reporting

Please select the one below that is the best fit for your research. If you are not sure, read the appropriate sections before making your selection.

☒ Life sciences ☐ Behavioural & social sciences ☐ Ecological, evolutionary & environmental sciences

For a reference copy of the document with all sections, see nature.com/documents/nr-reporting-summary-flat.pdf

Life sciences study design

All studies must disclose on these points even when the disclosure is negative.

Sample size	Sample sizes were determined using our previous experience with similar assays to provide sufficient data to calculate statistical significance. In most cases experiments were performed in biological triplicate or quadruplicate as indicated, which provided sufficient statistical power. For in vivo work, to calculate the sample size for each type of assay, a power analysis was performed using widely recognised statistical software (e.g., G*Power). A significance level of $\alpha = 0.05$ and a power $(1-\beta) \geq 0.8$ was set, using as reference an effect size estimated from our previous experience, preliminary laboratory data, and published values for MPXV infections. Using these calculations, group sizes of at least 5 male and 5 female mice were used.
Data exclusions	No data were excluded.
Replication	All experiments were performed 2-3 times as indicated to ensure reproducibility of the data. All repeat experiments gave the same result. The only exceptions are the RNA-seq experiments which were performed once in biological triplicates.
Randomization	All mice were randomly assigned to treatment groups. Plaques were selected at random for size quantification.
Blinding	All in vivo experiments were blinded. Investigators were not blinded to experimental groups for in vitro experiments as these required prior knowledge for data acquisition and interpretation.

Reporting for specific materials, systems and methods

We require information from authors about some types of materials, experimental systems and methods used in many studies. Here, indicate whether each material, system or method listed is relevant to your study. If you are not sure if a list item applies to your research, read the appropriate section before selecting a response.

Materials & experimental systems

n/a	Involved in the study
☐	☒ Antibodies
☐	☒ Eukaryotic cell lines
☒	☐ Palaeontology and archaeology
☐	☒ Animals and other organisms
☒	☐ Clinical data
☒	☐ Dual use research of concern
☒	☐ Plants

Methods

n/a	Involved in the study
☒	☐ ChIP-seq
☒	☐ Flow cytometry
☒	☐ MRI-based neuroimaging

Antibodies

Antibodies used

p-STAT1 (Tyr701) (58D6) 1:1000 Cell signalling technologies 9167S
STAT1 (D1K9Y) 1:1000 Cell signalling technologies 14994S
p-STAT2 (Tyr690) (D3P2P) 1:1000 Cell signalling technologies 88410T
STAT2 (D9J7L) 1:1000 Cell signalling technologies 72604T
p-STING (Ser366) (D7C3S) 1:1000 Cell signalling technologies 19781S
STING (D2P2F) 1:1000 Cell signalling technologies 13647S
p-IRF3 (Ser386) (EPR2346) 1:1000 Abcam ab76493
IRF3 (EPR2418Y) 1:1000 Abcam ab68481
p-ERK1/2 (Thr202/Tyr204) 1:1000 Cell signalling technologies 9101S
ERK 1:1000 Proteintech 66192-1-Ig
p-JNK (Thr183/Tyr185) 1:1000 Cell signalling technologies 9251S
JNK 1:1000 Cell signalling technologies 9252S
p-p38 (Thr180/Tyr182) 1:1000 Cell signalling technologies 9211S
P38 1:1000 Cell signalling technologies 9212S
DUSP1 (E6T5S) 1:1000 Cell signalling technologies 35217T
MxA (D3W7I) 1:500 Cell signalling technologies 37849S
IFIT1 (D2X9Z) 1:1000 Cell signalling technologies 14769S
FLAG (M2) 1:5000 Sigma F1804
β-Actin (2D4H5) 1:10000 Proteintech 66009-1-Ig
α-Tubulin (DM1A) 1:5000 Merck Millipore 05-829
rabbit-anti-OPG079 1:2000, a gift from D. Ulaeto, Defence Science and Technology Laboratory
rabbit-anti-OPG057 1:1000, a gift from D. Ulaeto, Defence Science and Technology Laboratory
Licor IRDye 800CW Donkey Anti-goat IgG secondary antibody (Licor, 925-3221 1:10,000 dilution, Licor IRDye 680CW donkey-anti-mouse IgG secondary antibody (Licor, 925-68072) 1:10,000 dilution.

Validation

The rabbit-anti-OPG079 serum was validated in-house using shRNA targeting OPG079.
The rabbit-anti-OPG057 serum was validated previously: PMID: 8648667.

Validation details for commercial antibodies used in this study can be found at:
rabbit-anti-pSTING: https://www.cellsignal.com/products/primary-antibodies/phospho-sting-ser366-d7c3s-rabbit-mab/19781
rabbit-anti-STING: https://www.cellsignal.com/products/primary-antibodies/sting-d2p2f-rabbit-mab/13647
rabbit-anti-pIRF3: https://www.abcam.com/en-us/products/primary-antibodies/irf3-phospho-s386-antibody-epr2346-ab76493
rabbit-anti-IRF3: https://www.abcam.com/en-us/products/primary-antibodies/irf3-antibody-epr2418y-ab68481
rabbit-anti-pSTAT1: https://www.cellsignal.com/products/primary-antibodies/phospho-stat1-tyr701-58d6-rabbit-mab/9167
rabbit-anti-STAT1: https://www.cellsignal.com/products/primary-antibodies/stat1-d1k9y-rabbit-mab/14994
rabbit-anti-pSTAT2: https://www.cellsignal.com/products/primary-antibodies/phospho-stat2-tyr690-d3p2p-rabbit-monoclonal-antibody/88410
rabbit-anti-STAT2: https://www.cellsignal.com/products/primary-antibodies/stat2-d9j7l-rabbit-monoclonal-antibody/72604
rabbit-anti-pERK1/2: https://www.cellsignal.com/products/primary-antibodies/phospho-p44-42-mapk-erk1-2-thr202-tyr204-antibody/9101
mouse-anti-ERK1/2: https://www.ptglab.com/products/ERK1-2-Antibody-66192-1-Ig.htm
rabbit-anti-pJNK: https://www.cellsignal.com/products/primary-antibodies/phospho-sapk-jnk-thr183-tyr185-antibody/9251
rabbit-anti-JNK: https://www.cellsignal.com/products/primary-antibodies/sapk-jnk-antibody/9252
rabbit-anti-pp38: https://www.cellsignal.com/products/primary-antibodies/phospho-p38-mapk-thr180-tyr182-antibody/9211
rabbit-anti-p38: https://www.cellsignal.com/products/primary-antibodies/p38-mapk-antibody/9212
rabbit-anti-DUSP1: https://www.cellsignal.com/products/primary-antibodies/dusp1-mkp1-e6t5s-rabbit-monoclonal-antibody/35217
rabbit-anti-IFIT1: https://www.cellsignal.com/products/primary-antibodies/ifit1-d2x9z-rabbit-mab/14769
rabbit-anti-MxA: https://www.cellsignal.com/products/primary-antibodies/mx1-d3w7i-rabbit-mab/37849
mouse-anti-tubulin: https://www.merckmillipore.com/GB/en/product/Anti-Tubulin-Antibody-clone-DM1A,MM_NF-05-829
mouse-anti-actin: https://www.ptglab.com/products/Pan-Actin-Antibody-66009-1-Ig.htm
mouse-anti-FLAG: https://www.sigmaaldrich.com/GB/en/product/sigma/f1804
Licor IRDye 800CW: https://shop.licor.com/bio/reagents/irdye-800cw-donkey-anti-goat-igg-secondary-antibody
Licor IRDye 680CW: https://shop.licor.com/bio/reagents/irdye-680rd-donkey-anti-mouse-igg-secondary-antibody

Eukaryotic cell lines

Policy information about cell lines and Sex and Gender in Research

Cell line source(s)	Human foetal foreskin fibroblasts (HFFF2, male) ECACC #86031405<br>BSC40 obtained from D Ulaeto, Dstl; ATCC #CRL-2761<br>U20S obtained from GL Smith, University of Oxford; ATCC #HTB-96<br>HEK293T obtained from GL Smith, University of Oxford; ATCC #CRL-3216<br>THP-1 IFIT-1 Gluc cells obtained from V Hornung, LMU Munich (Mankan, et al.EMBO J. 2014 Nov 25;33(24):2937–2946. doi: 10.15252/embj.201488726).
Authentication	We did not authenticate the cell lines used in this study.
Mycoplasma contamination	Cells were tested quarterly for mycoplasma contamination and were all negative.
Commonly misidentified lines (See ICLAC register)	None used.

Animals and other research organisms

Policy information about studies involving animals; ARRIVE guidelines recommended for reporting animal research, and Sex and Gender in Research

Laboratory animals	CAST/EiJ mice were used in this study, aged 7-12 weeks old. Mice were originally obtained from The Jackson Laboratory (Strain #:000928) and bred in-house.
Wild animals	Research did not involve wild animals.
Reporting on sex	Both male and female mice were used in this study as indicated.
Field-collected samples	This study did not involve field-collected samples.
Ethics oversight	All animal work was approved by the Ethical Review Board of CBM and CSIC under reference PROEX 241.1/21

Note that full information on the approval of the study protocol must also be provided in the manuscript.

Plants

Seed stocks	N/A
Novel plant genotypes	N/A
Authentication	N/A
